# Innovative Biobased and Sustainable Polymer Packaging Solutions for Extending Bread Shelf Life: A Review

**DOI:** 10.3390/polym15244700

**Published:** 2023-12-13

**Authors:** Vito Gigante, Laura Aliotta, Roberta Ascrizzi, Laura Pistelli, Angela Zinnai, Giovanna Batoni, Maria-Beatrice Coltelli, Andrea Lazzeri

**Affiliations:** 1 Department of Civil and Industrial Engineering, University of Pisa, Via Diotisalvi 2, 56122 Pisa, Italy; laura.aliotta@unipi.it (L.A.); maria.beatrice.coltelli@unipi.it (M.-B.C.); andrea.lazzeri@unipi.it (A.L.); 2 Department of Pharmacy, University of Pisa, Via Bonanno 6, 56126 Pisa, Italy; roberta.ascrizzi@unipi.it; 3 Interdepartmental Research Center “Nutraceuticals and Food for Health” (NUTRAFOOD), University of Pisa, Via del Borghetto 80, 56124 Pisa, Italy; laura.pistelli@unipi.it (L.P.); angela.zinnai@unipi.it (A.Z.); 4 Department of Agriculture Food Environment, University of Pisa, Via del Borghetto 80, 56124 Pisa, Italy; 5 Department of Translational Research and New Technologies in Medicine and Surgery, University of Pisa, Via S. Zeno 37, 56123 Pisa, Italy; giovanna.batoni@unipi.it

**Keywords:** biobased polymers, active biomolecules, shelf life, bread packaging

## Abstract

Sustainable packaging has been steadily gaining prominence within the food industry, with biobased materials emerging as a promising substitute for conventional petroleum-derived plastics. This review is dedicated to the examination of innovative biobased materials in the context of bread packaging. It aims to furnish a comprehensive survey of recent discoveries, fundamental properties, and potential applications. Commencing with an examination of the challenges posed by various bread types and the imperative of extending shelf life, the review underscores the beneficial role of biopolymers as internal coatings or external layers in preserving product freshness while upholding structural integrity. Furthermore, the introduction of biocomposites, resulting from the amalgamation of biopolymers with active biomolecules, fortifies barrier properties, thus shielding bread from moisture, oxygen, and external influences. The review also addresses the associated challenges and opportunities in utilizing biobased materials for bread packaging, accentuating the ongoing requirement for research and innovation to create advanced materials that ensure product integrity while diminishing the environmental footprint.

## 1. Introduction

As a result of rapid socio-economic development, bread (and, generally, bakery products) has gained widespread popularity, becoming an essential part of people’s daily diet worldwide [1]. To preserve bread quality, it is essential that the packaging maintains its properties for as long as possible, increasing the “shelf life”, i.e., the duration during which a product can be stored without losing its suitability for use, consumption, or sale [2]. Understanding the reasons behind physical, chemical, and microbial spoilage of bakery products in the food industry is crucial to prevent quality deterioration and economic losses for both manufacturers and consumers [3]. Indeed, the potential for microbial contamination and lipid and protein oxidation in bakery products also poses significant health concerns [4]. Therefore, ensuring the quality and safety of these products is paramount, and this heavily relies on the selection of appropriate packaging materials and technologies [5].

In this context, it is necessary to establish an innovative, resilient, productive, and, above all, sustainable bread-packaging economy by disconnecting the bread-packaging industry from fossil fuel resources and enabling the return of nutrients to the soil [6].

From these insights comes the need for a comprehensive review regarding the innovative biobased and sustainable polymer packaging solutions for extending bread shelf life. In detail, the objective of this review is to discuss the latest developments in bread-packaging biobased polymer systems, focusing on the diverse typologies of solutions and the major challenges that bread packaging must address soon to enter the virtuous loop of circular bioeconomy.

In fact, traditional packaging faces limitations in effectively safeguarding and maintaining the safety, hygiene, and quality of final products; indeed fossil-based plastics pose serious disposal challenges and have significant negative environmental impacts due to their non-biodegradable nature and dependence on non-renewable resources [7]. Nevertheless, advancements in modern technology enable packaging to interact with active/functional substances, enhancing its protective capabilities; innovative packaging is revolutionizing the way food items are packaged, introducing various methods to extend and monitor the shelf life of products [8]. Moreover, the toxic chemical components, such as flame retardants, pigments, and plasticizers, utilized in plastic packaging preparation can migrate and contaminate food, leading to adverse health effects [9].

In this context, biodegradable packaging or environmentally friendly alternatives, like biobased and compostable films and coatings, are gaining popularity for their ability to preserve bread while reducing environmental impact [10]. To meet the requirements of the eco-conscious consumer market and enhance product longevity, sustainable packaging solutions relying on biobased and/or biodegradable polymer systems must be viable.

The current review is organized to start with a comprehensive overview of common issues associated with bread degradation and the current packaging technologies employed to pack the bread. Subsequently, it will delineate the challenges involved in developing innovative, sustainable, biobased systems specifically designed for packaging bread with the goal of prolonging its shelf life. The overall objective is to highlight the advantages and constraints of biobased polymers used in bread packaging in comparison to traditional materials. The analysis will also encompass the role of active biomolecules that can be integrated into biopolymeric matrices to enhance the shelf life of bread products. Furthermore, the review will explore existing market applications, underscore the significance of aligning innovative materials with sustainable design principles, and discuss future trends, including legislative and economic obstacles that need to be overcome in this field.

Therefore, summarizing, in our opinion there is a need to collect updated knowledge about the biobased polymeric systems with the addition of active biomolecules capable of providing specific properties to the packaging and increasing the shelf life of bread. Certainly, in the literature, there are several review works, including recent ones, concerning the issues related to ensuring good packaging for bread [11,12], but there is a lack of information regarding biobased and sustainable solutions that address this matter.

## 2. The Bread: Characteristics and Problems Related to the Packaging

### 2.1. Main Features and Characteristics of the Bread Typologies

The accelerated growth in the bread product industry can be attributed to the increased availability of consistently high-quality raw materials, the introduction of novel bakery products, and cost reduction measures [13]. Typically, bread is a baked product comprising a simple combination of ingredients, namely flour, water, salt, and yeast [14]. The transformation from a mixture of these ingredients and other functional elements into the final baked loaf of bread is often likened to a shift from a foam to a sponge. In a foam, gas bubbles introduced during mixing remain separate, encapsulated by the gluten network, while in a sponge, the gas cells are open and interconnected. This transition is responsible for the distinctive cellular structure of bread [15].

The process of bread production involves a series of biochemical and physicochemical transformations that influence the characteristics of the final product [16]. Notably, water and flour stand as the primary constituents in a bread recipe, exerting the most substantial influence on its texture and crumb structure [17], while the flavor of bread is influenced by the baking process, which leads to the formation of a colored crust as a result of the Maillard reaction, as well as the development of acidity within the crumb due to fermentation [18].

Cauvain et al. [19] stated that the primary differentiation in various bread-making methods lies in the way dough ingredients are mixed and how the gluten network is developed, categorizing the main bread-making processes into four primary groups: (i) straight dough bulk fermentation; (ii) sponge and dough; (iii) rapid processing; (iv) mechanical dough development. Each of these processes within the primary bread-making groups tends to yield subtly different characteristics in the end product, which, in turn, has implications for the shelf life and stability of the bread.

Generally speaking, shelf life of bread kept at room temperature ranges from 3–7 days but may vary depending on ingredients, type of bread, and storage method. It is also pertinent to emphasize the significance of production conditions and the reactions occurring during the bread-making process, which convert the initial raw ingredients into the final product. Each stage contributes to a certain degree to the ultimate rheological and flavor characteristics of the bread product and, consequently, its shelf life [20].

For instance, many commercially available bread products such as sandwiches, loaves, and bakery breads are frequently enriched with preservatives to ward off mold formation and extend their shelf life [21]. In the absence of preservatives, conventional bread typically remains fresh for 3 to 4 days when stored at room temperature. In contrast, gluten-free bread, due to its elevated moisture content and limited use of preservatives, is more prone to mold development [22]. This is why it is commonly distributed in a frozen state rather than at room temperature [23]. Conversely, dried bread items like breadcrumbs and crackers tend to have a longer shelf life as mold growth necessitates moisture [11].

Definitely, bread shelf life is primarily governed by the onset of staling and microbial spoilage-induced ropiness [24]. Additional factors impacting shelf life include rancidity, crystallization, grittiness, syneresis in jams and jellies, the development of undesirable flavors and odors beyond rancidity, structural degradation, fading color, and moisture migration. To prolong the shelf life of bakery products, it is imperative to employ effective preservation methods that address these factors [25]. Subsequent paragraphs will delve into this subject in greater detail.

### 2.2. Current Packaging Technologies Employed for Bread Products

**Figure 1:**
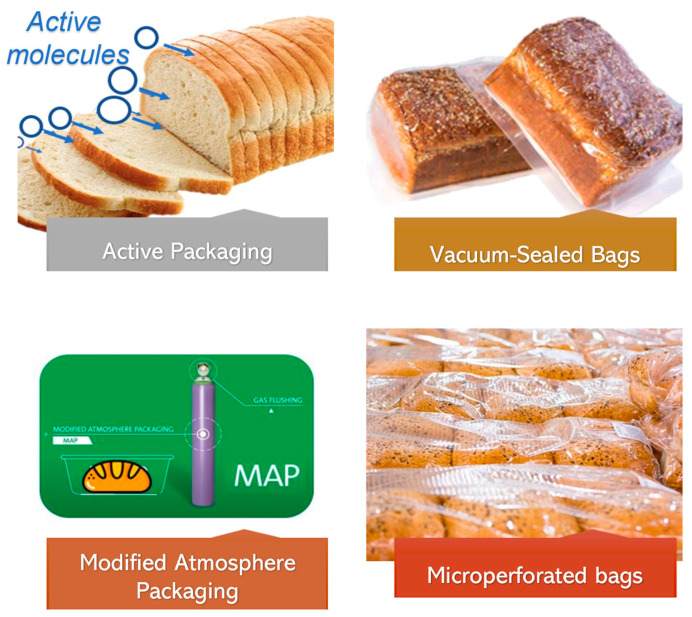
Typical technologies to pack bakery products.

Various are the bread-packaging solutions. Among these solutions, also shown in Figure 1, bread-based products can be packaged, for example, in a modified atmosphere through modified atmosphere packaging (MAP) considering the control of oxygen, carbon dioxide, and nitrogen levels within the packaging to effectively slow down the staling process of the product and prevent microbial growth [26]; in it, CO_2_′s fungistatic properties disrupt the growth of mold by altering the metabolic activity of the cell membrane, leading to a detrimental impact on its growth [27]. Bread packaging can also be vacuum-sealed to remove air from the package, creating an oxygen-free environment; this reduces the potential for lipid oxidation and microbial contamination, effectively extending the bread’s freshness [28]. It is also possible to package bread with the flow pack method using microperforated bags: these packaging bags have small perforations that allow for controlled airflow while limiting excess moisture, striking a balance between maintaining optimal moisture levels and preventing mold growth [25]. Another solution frequently used is the possibility to cover a polymer surface by applying sustainable coatings, creating a protective layer that prevents the migration of moisture and gases with improved and multifunctional performances [29]. Indeed, during bread storage, microbial changes primarily impact the quality and consumers’ acceptance, necessitating the use of antimicrobial packaging. By incorporating antimicrobial agents into packaging materials, the growth of harmful microorganisms can be inhibited, ensuring the safety and quality of the bread [30]. It is also important to prevent the formation of mold, whose formation is, indeed, influenced by a combination of extrinsic and intrinsic factors, encompassing temperature, rainfall, humidity, and light as extrinsic factors and oxygen availability, preservatives, the pathogen’s behavior in the food matrix, and water activity as intrinsic factors [31]; in this context, active packaging systems incorporate additives, such as oxygen scavengers or moisture absorbers, which are useful to maintain a desirable environment within the package and extend shelf life [32].

Other solutions are also the combination of both traditional systems and intelligent packaging, for example, it has been demonstrated that combining modified atmosphere packaging (MAP) with active packaging enhances bread shelf life, nutritional characteristics, and safety of the products [33].

### 2.3. Volatile Compounds Involved in Bread Aroma: Their Development and Packaging-Related Problems

Bread aroma is of the utmost importance in driving consumers’ preference: as a widespread product, its flavor is a key attribute to consider during the processing and storage phases. The former is fundamental for the development of the involved molecules, while the latter must ensure the retention of these volatile compounds and avoid the production of off-flavors due to spoilage.

Over 540 volatile organic compounds (VOCs) have been identified in bread, with a focus on different recipes and parts (crust and crumb) [34]. Wheat flour’s contribution to flavor appears minimal, but subsequent processing phases play a crucial role in developing the bread aroma bouquet [35]. Flour hydration and mixing trigger enzymatic activities, including amylases, proteases, and lipoxygenases, crucial for flavor development [36]; these enzymes contribute to the formation of precursors, such as peptides and free amino acids, which transform into aroma-active compounds during baking [37]. Yeasts and lactic acid bacteria in the fermentation phase generate key compounds for bread aroma, especially in the crumb [38,39]. Baking induces sugar caramelization, non-enzymatic Maillard reactions [40], and the absorption of flavor molecules during the cooling phase; moreover, volatiles absorbed during cooling evaporate mainly from the crust, leading to a fast dissipation of bread aroma [13]. Finally, bread aroma undergoes changes during storage, with a decrease in desirable roasty, malty, and sweet notes and an increase in fatty off-flavors due to lipid oxidation when staling off-flavors begin to be perceived [41,42].

While the effect of different packaging techniques to prolong the shelf life has been widely studied regarding microbiological stability and physical properties, their effect on bread aroma needs to be explored.

Some alternative modified atmosphere packaging (MAP), such as the inclusion of oxygen scavengers in sachets, can reduce the development of oxidation-induced off-flavors [43]. Antimicrobial compounds have also been formulated in sachets to be added to bread, such as eugenol and citral, which exerted a synergistic inhibition against *Aspergillus niger* without affecting the sensory attributes of bread [44]. However, the presence of a sachet is not consumer-friendly for safety reasons (i.e., risk of accidental ingestion) and has higher costs [43]. Another MAP technique uses ethanol emitters in the headspace of bread: however, while it successfully retards the growth of spoilage microorganisms, it could induce the generation of off-flavors [45]. Moisture-absorbing sachets are also used in baked product packaging [46]: the control of the relative humidity of the final product is important to avoid the modification of the partition coefficients of the volatiles between the headspace and the matrix, thus avoiding the loss of the desirable aroma notes from the bread [40].

### 2.4. Factors Affecting Shelf Life of Bread and Bakery Products

The spoilage of bread and bakery products is directly correlated to their shelf life and it is affected by molds, bacteria, and yeasts [47]. Molds are the major spoilage problem. These microorganisms are a type of fungus that consists of multiple cells in contrast to yeasts, which are single celled. Mold proliferation is favored by the presence of water, thus appearing frequently in bakery products with a high-water activity level such as cakes and creams. *Rhizopus stolonifera*, *Penicillium*, and *Aspergillus* are well-known species. Some molds are more hazardous to mankind when consumed, as they produce mycotoxins [48].

Bacteria also have the potential to contaminate baked products. The spores of *Bacillus subtilis*, for example, are heat resistant and will not be killed by the baking process. *Staphylococcus aureus* is one type of bacteria known to contaminate pie fillings. In the European Union, around 100,000 human cases related to *Salmonella* (e.g., *Salmonella enteritidis*) are reported each year by the EFSA, 4% are dedicated to bakery products according to a report of the EFSA published in 2014 [49].

Problems caused by yeasts can be divided into two types: visible yeasts which grow on the surface of the bread and fermentative spoilage, associated with alcoholic and essence odors and hence osmophilic yeasts. Food producers want to prevent or delay mechanisms of food deterioration and spoilage to reduce costs, ensure food safety, and extend shelf life, while maintaining food quality. Usually, three techniques are considered: (i) inactivation of microorganisms; (ii) prevention or inhibition of microbial growth; (iii) restricting the access of microorganisms to products [50].

**Figure 2:**
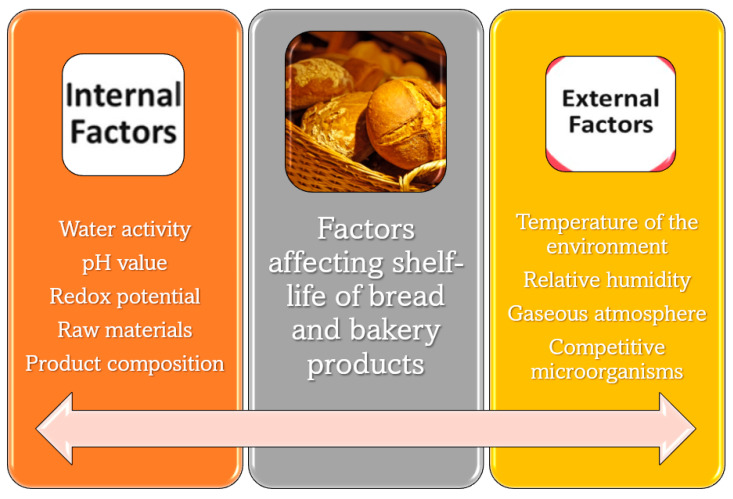
Internal and external factors affecting shelf life in bread and bakery products.

Impacting on mold, bacterial, and yeast proliferation, several physical chemical internal or external factors (Figure 2) influence shelf life of bread and bakery products [51]. Internal factors are the characteristic of the product, including its water content, its pH value, and its redox potential (influencing its behavior to oxidation). Nutrients contained in raw materials can support the growth of microorganisms. The composition, including specific additives, can much affect the shelf life. For instance, fats and salts generally induce an increase in shelf life. In microbiology, four major groups of microorganisms are classified based on their temperature ranges for growth, from the highest to the lowest: thermophiles, mesophiles, psychrophiles, and psychotrophs. Mesophiles, with optimal temperature around 37 °C, include many of the common foodborne pathogens [52].

Temperature is a fundamental external physical–chemical factor affecting bread and bakery spoilage. Microorganisms have their specific range of temperature where growth is accomplishable. In particular, microorganisms are not able to grow at temperatures below −8 °C and above 100 °C. Bacteria are normally limited to a temperature span around 35 °C and molds around 30 °C. Baking, chilling, and freezing prevent or limit microorganism growth. The relative humidity of the environment interacts with the relative humidity inside the product. Moisture in bakery products and its immediate environment will always transfer from high to low relative humidity [53]. Regarding gaseous atmosphere, the typical air atmosphere allows microorganism proliferation, whereas a modified atmosphere can inhibit microorganism growth. Moreover, in this context, the gas barrier properties of packaging can play a very important role, as evidenced by Licciardello et al. [54]. The presence of competitive species, for instance, antibiotics, can play a role in preventing microorganism growth. Controlling all these factors through suitable technologies in food processing is fundamental to grant product quality, safeness, and a long shelf life. The use of different kinds of physical–chemical, image, and sensorial analysis allows obtaining complementary results, which help to draw general conclusions on the overall quality loss of a bakery product [55].

### 2.5. Problems to Overcome for Slowing the Spoilage

**Figure 3:**
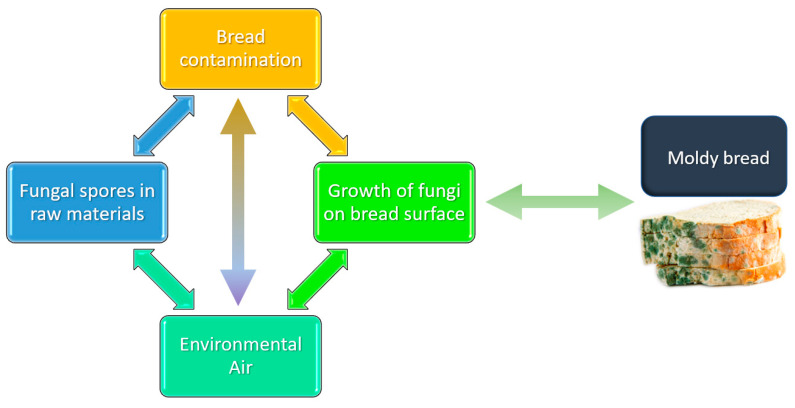
Fungal contamination of breads in industry.

Generally, contamination originates predominantly in the post-baking step by fungal spores being deposited from the bakery environment. Oxygen, temperature, pH, and water are the parameters mainly affecting this process [23] as also evidenced in the previous paragraph. Additionally, plant pathogens during cultivation and fungal contamination in the post-harvest, storage, and pre- and post-processing stages can introduce microorganism contamination in bread. In the bakery chain, the air is described as one of the principal sources of contamination (Figure 3). In fact, the spores present in the industrial processing environment may recontaminate the food after baking, which mainly occurs in the slicing and packaging steps. The hygienic–sanitary conditions of the production environment and time that the bread is exposed to environmental air after its removal from the oven are also relevant factors that influence fungal load.

The ineffectiveness of traditional synthetic preservatives due to the development of resistance by several fungi increased the pressure to find alternative agents. In general bakery practice, molds do not survive the bread-baking process, but mycotoxins produced by crop pathogens and food spoilage fungi are relatively heat stable. The latter represents an important issue in the cereal chain worldwide, especially in climatic regions where the humidity and temperature are higher. Weak organic acids, such as propionic and sorbic acid, are commonly added as traditional chemical preservatives because they suppress the growth of undesired microorganisms [56]. Generally, easy to handle potassium, calcium, or sodium salts are used. Nevertheless, high concentrations of sorbate or propionate can also alter the organoleptic and sensory properties of the product.

Alternative preservation routes, considering biotechnology, can be considered. Fermentation of lactic acid bacteria (LAB) [21] can be exploited because it results in the production of acetic and lactic acid, suppressing fungi. Lactic acid bacteria were also explored for their effectiveness versus mycotoxins, but more work is required for better exploitation of this biotechnology.

The application of yeasts other than baker’s yeast (*Saccharomyces cerevisiae*) is also suggested as a promising alternative for bread preservation. Moreover, the use of plant extracts and essential oils for controlling common food spoilage fungi was explored: raisin extracts, cherry laurel leaf extracts, antifungal proteins from vegetables (for instance, from Amaranthus seed extracts) or microorganism sources, and water/salt-soluble extracts of different legume flour hydrolysates (soy, lentil, pea, chickpea, and faba bean) were considered [57]. The use of biosurfactants or biopreservatives derived from plants is frequently proposed as an alternative to synthetic preservatives. Antifungal proteins were explored as well as antimicrobial essential oils, that could also contribute to an improved sensory profile.

Predictive microbiology (or microbial modeling) is the use of mathematical models or equations to predict in a food system the growth and/or activity of a microorganism as a function of time. With the development of predictive mycology, models have been developed to describe mainly fungal germination and the inactivation of these microorganisms [48]. Predictive methods could be important tools to extend the shelf life of bread and bakery products.

Incorporation of volatile antimicrobial substances from spices and herbs into packaging materials may provide alternative effective ways of prolonging the shelf life of bread without the use of synthetic preservatives. A combination of allyl isothiocyanate (AITC) and sorbate may also solve this problem [12]. Isothiocyanates are a group of potential antimicrobial substances. They occur naturally in cruciferous vegetables, such as broccoli, cabbage, cauliflower, kale, turnip, radish, canola, rapeseed, and various mustards. They could be effective in reducing aflatoxins in bread [58].

To improve shelf life, physical treatments such as UV, MW, or IR irradiation resulting in an antimicrobial action can also be considered. Lipopeptides produced by microorganisms have attracted the attention of the food industry due to their antimicrobial activity. The addition of ethanol is also a traditional preservative method. Sourdough fermented with antifungal strains showed high potential to produce breads with an extended shelf life but also an improved nutritional value, bread quality, and safety [59].

## 3. Active Biomolecules for Improving Bread Shelf Life

In the following subsections, the review will describe the state of the art regarding the potential biomolecules that can be utilized to impart specific properties to sustainable packaging, including oxygen barrier, water vapor barrier, antimicrobial properties, antioxidants, and molecules capable of acting as preservatives for bread.

### 3.1. Oxygen Barrier

In food conservation, it is necessary to consider that oxygen is highly reactive because it can oxidize some components of foods (for example, causing fats to oxidize, making the food rancid).

The introduction of oxygen barrier layers in packaging is generally carried out if the bakery product’s shelf life needs to be increased. The polymers with greatest oxygen barrier properties, mainly poly(ethylene-co-vinyl alcohol) (EvOH) and poly(vinylidene chloride) (PVDC), have poor mechanical properties and are very sensitive to external agents. In fact, the former is sensitive to water, thus it is mainly present as an intermediate layer in multilayer packaging in which external polyolefin and polyester layers guarantee a barrier to water vapor and CO_2_, respectively, depending on the specific food to pack. The latter is sensitive to temperature which may cause the bonds between carbon and chlorine to break, resulting in a decrease in the transparent appearance of the material, ideal for packaging, and making it brown. Increasing the oxygen barrier properties of plastic film is a challenge of current research. The use of oxygen barrier packaging containing EVOH layer in fossil-based plastic like polyethylene and polyamides was considered a correct strategy for bakery products [60].

Interestingly, several biobased polymers were efficient in increasing the barrier properties of plastic films and cellulosic substrates. The intrinsic hydrophilicity of protein films results in good adhesion to polar surfaces, such as paper or paperboard, and a good barrier to apolar gases such as oxygen and carbon dioxide but not water vapor [61,62]. Thanks to their good oxygen barriers at low relative humidity, various proteins have been tested and used as edible coatings/films such as those from gelatin [63], casein, whey, corn zein, wheat gluten [64], and peanut. In addition, some high-molecular-weight proteins are insoluble in water, thus they may be used to obtain water-resistant films [65]. Gelatin-based coatings on PE, PET, and PP improved barrier properties of substrates against oxygen and also UV radiation [66]. Trezza et al. showed that zein-coated paper had better oxygen barrier properties than PE (barrier improvement factor up to 73) so they suggested that they can applied on paper packaging as an alternative to paraffin [67]. Plasticized corn zein coating (having a thickness of 3–4 μm) applied on PP films resulted in a reduction in oxygen permeability of three orders of magnitude [68]. In addition, when combined with a protein-based coating, biodegradable substrate materials maintain their ability to biodegrade [69]. Polysaccharides were also considered to develop coatings for improving the gas barrier properties of plastic and bioplastic films as well as cellulosic substrates [70]. Nanocellulose was recently explored for the production of coatings for fossil or biobased substrates or biocomposite films [71]. Coatings made of plasticized nanocellulose cross-linked with sorbitol and citric acid were produced for improving paper-based packaging. The synthesized nanocellulose was dip coated on paper. It was found that the toughness, barrier properties, and thermal stability were enhanced. The oxygen permeability value was found to be low (0.7 mL μm day^−1^ m^−2^ kPa^−1^ at 49% RH) and it had thermal stability up to about 300 °C. The oxygen permeability was found to be better than other polymer samples like polystyrene (PS), PLA, and polyethylene terephthalate (PET). Moreover, the water vapor permeability was reduced by 60% [72]. In biocomposite films, the dispersion of nanocellulose by extrusion was reported to improve PLA gas barrier properties, without, however, reaching standard food-packaging requirements [73]. Thus, multilayer packaging was proposed by Guivier et al. [74] considering a PLA film layer and a nanocellulose coating. Corona treatment and chitosan were used to improve the adhesion of nanocellulose to PLA. The multilayer packaging was efficient in decreasing the oxygen permeability of a PLA film by two orders of magnitude. Chitin is the second most abundant polysaccharide after cellulose. In fact, it represents the structural polymer of arthropods (including crustaceans and insects), mollusks, and several mushrooms [75]. After demineralizing and deproteinizing, biomasses are converted into chitin [76]. Chitin can be modified into chitin nanofibrils, representing its crystalline part, or chitosan [77] though a partial or complete deacetylation, respectively. Chitosan and chitin nanofibril coatings were found to improve oxygen barrier properties of both cellulosic substrates [78] and bioplastic films [79], thus suggesting an efficient strategy to obtain packaging that is fully renewable and compostable. Hence, these coatings can be particularly useful to reduce oxygen permeability for bread and bakery products as assessed by Bhardwaj et al. [80] who tested the consumer acceptance and storability of chitosan and beeswax-coated cellulose packaging for whole wheat bread. Ji et al. [81] applied chitin nanowhisker-based coating onto cellulose acetate films and, after optimizing the formulation and coating process, they obtained a reduction of oxygen permeability of 91–99% thanks to the applied coating.

In all the biopolymers described above, strong intermacromolecular hydrogen bonds are present. Thus, apolar molecules like O_2_ and CO_2_, on one hand, have a poor chemical affinity to the highly polar proteins and polysaccharides, and, on the other hand, struggle to diffuse through the biopolymer film due to the existence of an enormous number of hydrogen bonds between the macromolecules [82].

### 3.2. Water Vapor Barrier

Polyolefin films are considered the best for granting a reduced water vapor permeability in food packaging. However, renewable, recyclable, and compostable alternatives should replace them in the future [83]. Biopolyesters are a class of polymers that can be produced on a wide industrial scale from renewable sources. Among these polymers, starch-based materials and poly(lactic acid) (PLA) are the most widely used and cheap. However, other polyesters like polybutylene succinate (PBS) and its copolymers and poly(butylene adipate-co-terephthalate) (PBAT) are also almost completely renewable and compostable. These polyesters generally show slightly higher water vapor permeability than polyolefins, but it is still low enough to grant a water vapor barrier suitable for bakery products. In these products, water vapor should not diffuse out from the packaging, otherwise the product may become dry and lose its organoleptic properties [84]. Interestingly, poly(hydroxyalkanoates) (PHAs) are a class of polymers produced by bacteria, compostable in several environmental conditions (included marine environments), and with enhanced oxygen and water vapor permeability. These good barrier properties are attributed at the high crystallinity of some PHAs [85]. The production of blends of different biopolyesters can be an additional strategy to modulate properties, as well as the production of biocomposites and multilayer systems [86].

Coatings based on natural waxes can also be considered to improve films’ hydrophobicity, resulting in an enhanced water vapor barrier. In particular, beeswax was used by Spotti et al. [87] in edible polysaccharidic films to enhance their hydrophobicity. Soybean oil was also used to prepare packaging for bakery products [88]. The presence of vegetable oil in a formulation based on corn starch and methylcellulose decreased the water vapor permeability of the packaging.

Cutin is a polymer present in the peel of fruits and vegetables and it consists of a cross-linked polyester [29]. Cutin can be extracted by depolymerization (cleavage of the ester bonds) using alkaline hydrolysis. The method has been patented and a pilot plant for cutin production is currently exploiting it [89]. Moreover, tomato cutin was used in combination with sodium alginate and beeswax in a green solvent (i.e., ethanol and water) to obtain a hydrophobic biopolymeric film [90]. Interestingly, Manrich et al., to confer hydrophobicity to packaging wraps, combined cutin with pectin [91]. All these studies suggested that cutin can be used as an additive, in polymeric formulation or coatings, to improve the hydrophobicity of packaging and, reasonably, the permeability to water vapor should be decreased [92].

### 3.3. Antimicrobial Properties

Prevention of microbial contamination and growth plays a major role in food packaging, contributing to avoiding deterioration and extending the shelf life of foodstuffs [93]. Bread after baking is usually free of bacteria and mold, but due to the relatively high content of water (approximately 40%), it may be easily contaminated by environmental mold following exposure to air during cooling, packaging, or storage. The most common spoilage microorganisms of bread and other bakery products are molds belonging to the genera *Eurotium*, *Aspergillus*, and *Penicillium* [94]. They may reduce the shelf life to 3–4 days at room temperature, in the absence of preservatives. Among bacteria, a concerning source of spoilage is represented by the heat-resistant spores of *Bacillus* species (e.g., *B. subtilis*, *B. licheniformis*, *B. pumilus*, and *B. cereus*) that may survive the baking process and, once germinated, result in the rope formation of bacteria, which cause a sticky texture and a fruity odor in bread [95]. Indeed, microbial contamination of bread may result not only in an unpleasant appearance but also be responsible for off-flavor and odor formation, alteration of bread texture, and possible production of microbial-derived toxins and allergenic compounds which may be present even before growth is visible [96]. Thus, besides reducing huge economic losses, prevention or delay of bread microbial contamination is of paramount importance to ensure consumers’ health.

**Figure 4:**
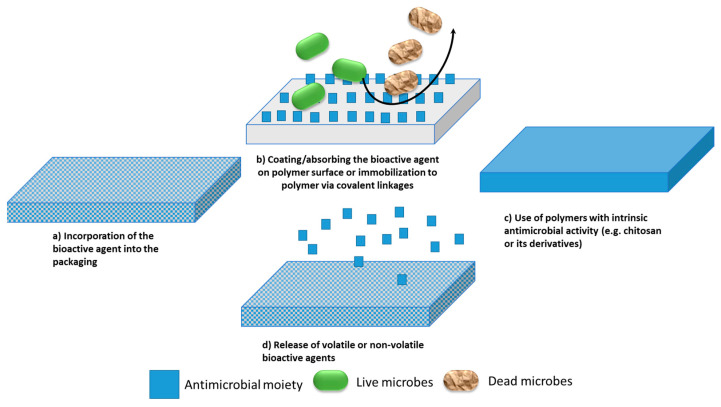
Overview of different strategies to confer antimicrobial properties to food packaging.

Several methods can be exploited to confer antimicrobial properties to food packaging (Figure 4). These essentially fall into two main categories: (i) systems that act by direct contact with the spoilage organisms without implying an active release of the antimicrobial moiety (Figure 4a–c); (ii) systems, also referred to as “controlled release packaging” [97], that release the antimicrobial moiety over extended periods of time to maintain the quality and the safety of foods (Figure 4d).

A third strategy is currently emerging as an innovative evolution of these systems. Referred to as “responsive packaging”, it consists in systems able to react to a stimulus present in the food or the environment [98]. In this case, the release of the antimicrobial compound is not continuous but can be triggered by chemical, biochemical, or biological changes occurring in the food product or the package environment following the biological activity of specific microorganisms. Although implementation of responsive material technology in food packaging is still in its infancy, this strategy could have a great impact on food safety in the years to come [99]. In general, antimicrobial agents can interfere with microbial growth by several mechanisms including destabilization of bacterial membranes, inhibition of replication, transcription, and translation of nucleic acids, alteration of the structure of key proteins through modification or denaturation, interference with cellular metabolism and enzymes involved in cell wall synthesis, and generation of reactive oxygen species. When incorporated in active food packaging, antimicrobial agents can alter the structure and the properties of the packaging materials including the mechanical strength, gas permeability, and optical, thermal, morphological, and physical properties, making the monitoring of all these characteristics mandatory when innovative antimicrobial substances are tested.

Antimicrobials are compounds or materials that significantly reduce the proliferation of bacteria, fungi, and mold. Common synthetic antimicrobial chemicals used in food, active towards bacteria and mold, are organic acids and their salts, sulfites, nitrites, antibiotics, and alcohols. Organic acids such as sodium benzoate and potassium sorbate are the most widely used preservatives [100]. Potassium sorbate was the most effective preservative to be used in bakery products. Currently, natural antimicrobial agents coming from biobased materials are preferred. Allyl isothiocyanate, a compound extracted from mustard and other plants, is also known as a good antimicrobial [101].

Natural antimicrobial compounds are reported to inhibit microbial growth in solution, on culture media, or on a variety of foods. Organic acids are added to food, but they are generally too polar to modify polymeric packaging for foods [102].

Phenolic acids, polyphenols, flavonoids, tannins, quinones, coumarins, terpenoids, and alkaloids are the major classes of compounds showing an antimicrobial action. Several naturally occurring phenolic compounds that are present in various herbs (garlic, oregano, thyme, and rosemary), plant sources such as fruits (apple, grape, pomegranate, and orange), vegetables (cabbage and onion), and spices (pepper, cardamom, and clove) showed antimicrobial properties [103]. Rosmarinic [104], ferulic, and caffeic acids extracted from plant or food waste can thus be used as additives for developing antimicrobial packaging. However, it is necessary to consider the necessity of controlling an efficient but modulable release of these beneficial additives. Often, the incorporation of these additives in inorganic nanostructured materials can be a correct strategy to achieve this goal.

The efficacy of these natural antimicrobials has been demonstrated in the laboratory. Nevertheless, their effectiveness in food packaging should be better explored. Other additives that should be mentioned are bacteriocins (nisin and lacticin), seed extracts, spice extracts (thymol, p-cymene, and cinnamaldehyde), enzymes (peroxidase and lysozyme), honey, and propolis extract. Essential oils are also strong antimicrobials (see Section 3.5), but generally they are lipophilic, hence they may not be concentrated in the aqueous media of food where microbial growth occurs [101].

In the food industry, the possible use of antimicrobial biobased polymeric systems is a growing trend, stimulating studies aimed specifically at exploring such systems as packaging to inhibit microbial growth and prolong the shelf life of bread and other bakery products [101]. For instance, recently poly(vinyl alcohol) (PVA) films functionalized by the incorporation of lignin were tested for their ability to inhibit mold growth on bread [105]. Lignin was synthesized from the waste leaves of *Ficus auriculata* obtained after the extraction of gallic acid. By virtue of its high content of phenolic moieties and other functional groups, lignin displays antioxidant and antimicrobial properties that make it an interesting element for food packaging [106]. Incorporation of lignin into PVA films caused a slight browning of the films that, however, retained sufficient transparency and an increase in tensile strength of the films, possibly due to the characteristic rigidity of lignin [105]. Interestingly, when progression of spoilage of bread packed with lignin-incorporated PVA films was analyzed, a net retardation of visible mold growth was observed as compared to commercial packages (3–4 days versus 22 days). Of note, the maximum retarding effect was obtained with PVA films added with 1% lignin, while when the lignin content was increased to 3% and 5% the antifungal performance of the films worsened. This finding highlights that several factors can contribute to the antimicrobial properties of a package that go beyond the content of the antimicrobial moieties and may involve other characteristics with impact on microbial growth such as oxygen or water permeability. In another study, pectin was evaluated as a polymeric matrix for the preparation of edible films incorporated with essential oils (EOs, Section 3.5.1), i.e., cinnamaldehyde (cinnamon oil) and eugenol (clove oil), as antimicrobial additives [107]. Pectin, one of the main components of plant cell walls, is considered a suitable polymeric matrix for the preparation of films for food packaging as it is biodegradable, biocompatible, edible, and exhibits suitable chemical and physical properties such as gelation and selective gas permeability. Incorporation of EO into pectin-based films resulted in transparent and homogeneous films, although slightly yellowish, with an increased tensile strength and tearing resistance as compared to control films. Incubation of moist bread with EO-enriched films at 30 °C increased the shelf life of 4 days, retarding the growth of *Penicillium* and *Aspergillus* [30]. Cinnamaldehyde (CIN) was also used as an antimicrobial additive to prepare poly(lactic acid)/konjac glucomannan/wheat gluten bilayer films (PLA/KGM/WG) consisting in PLA as the outer layer and CIN-loaded KGM/WG as the inner layer [108]. Microbiological analysis of homemade bread samples demonstrated a net increase in the total fungal count (from less than 1 log CFU/g at day 0 versus about 6 log CFU 7 g at day 10) in samples exposed to air. A very weak antifungal activity was observed in bread samples packed with PLA/KGM/WG films as compared to samples without film protection. In contrast, CIN-loaded PLA/KGM/WG films exhibited a strong antifungal effect decreasing the viable fungi count up to 5.8 CFU/g at the end of the observational period. An interesting aspect was disclosed by the study of Wang and coworkers that may have important implications for the food industry. The authors found that the antifungal efficacy of PLA/KG/WG-CIN films might vary according to the fermentation time of the homemade bread, being the highest in bread fermented for 200 min, followed by the bread fermented for 130 min and the bread fermented for 80 min [109]. This finding may be related to the possible effect of the fermentation time on microbial diversity of bread as well as on the structural properties of the final bread product (volume, microstructure, pore size) that, in turn, can impact the CIN absorption of bread and its antimicrobial activity. Long fermentation times may provoke yeasts to consume the nutrients (e.g., water, sugar, and proteins), causing a decrease in nutrients essential for subsequent fungal growth. Likewise, long fermentation times may yield bread products with a large pore size that may favor the diffusion and the interaction of bioactive molecules with microbial cells.

Chitin nanofibrils and chitosan biopolymers deserve to be mentioned in this section because of their antimicrobial properties, probably attributable to the presence of protonated -NH_2_. Chitin nanofibrils and chitosan induced antimicrobicity in paperboard-based packaging and resulted in the improvement of shelf life for fresh pasta [110]. Moreover, chitin nanofibrils present in a coating, made the surface of bioplastics antibacterial against *Staphylococcus aureus* and *Escherichia coli* [111].

From the few examples reported above, it can be inferred that the use of biobased polymeric systems to prevent microbial growth and extend the shelf life of bread looks promising, but the field is not free of possible pitfalls [112]. For instance, large-scale use of antimicrobials in active packaging could have a deleterious effect on the development of microbial resistance, an aspect that needs to be carefully taken into account when choosing new antimicrobial additives. Likewise, it is crucial to evaluate the type of microbes involved in the spoilage of specific food products in order to properly match the releasing kinetics or mode of action of the bioactive moieties with the time of growth of contaminants. Antimicrobials highly compatible with the polymeric matrices could be released too slowly to block the growth of fast-growing contaminants, while, vice versa, incompatible additives will be released within minutes and ineffective against slow-growing microbes [113]. More dedicated studies are therefore needed involving multidisciplinary teams that bring together experts from the fields of microbiology, food technology, engineering, and material science to ensure an effective and safe use of antimicrobials in the food-packaging industry.

### 3.4. Antioxidant Properties

**Figure 5:**
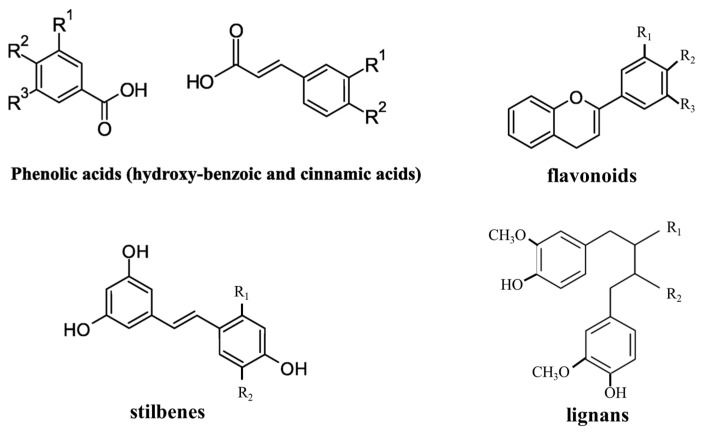
Different classes of polyphenols.

Primary and secondary antioxidants can be distinguished [114]. The former are essentially free-radical scavengers. In this class, the synthetic butylated hydroxy anisole (BHA), butylated hydroxy toluene (BHT), and tertiary butyl hydroquinone (TBHQ) [115] are included but, nowadays, due to their harmfulness to human health, natural compounds, including plant extracts and essential oils, are generally preferred. Plant extract-based natural antioxidant packaging can release the active compounds to protect foods from free radicals, intermediates, and secondary breakdown products. Moreover, these compounds can protect food because they integrate good antimicrobial and antifungal properties. Three subclasses can be considered to classify natural antioxidants: (i) vitamins (for instance, vitamins C and E), (ii) carotenoids (for instance, carotenes and xanthophylls), and (iii) polyphenols (for instance, flavonoids, phenolic acids, lignans, and stilbenes), illustrated in Figure 5.

Regarding the antioxidant properties of plant extracts, composition features determine the antioxidant effectiveness. Hence, the adopted extraction process (methodology, temperature, stirring efficiency, etc.), affecting the yield of extraction of the different compounds present in the extract, significantly affects the final antioxidant properties.

One important feature is the solubility in water. In fact, smaller and polar molecules, like flavonoids and phenolic acids, behaving as weak electrolytes, are generally more water soluble than larger lignans or stilbenes. Hence, they are more interesting for their antioxidant properties. Glycirrhetinic and rosmarinic acid [116] were added as antioxidant and antimicrobial additives in renewable and biodegradable biopolyester blend films consisting of polylactic acid (PLA) and poly(butylene adipate-co-terephthalate) (PBAT) for packaging. The release behavior as well as antioxidant and antimicrobial properties were tested and interesting differences were evidenced so a promising strategy for modulable active packaging was assessed.

Secondary antioxidants include: chelators that can be synthetic like EDTA or polyacrylic acid, but citric acid or lactoferrin can also be effective; UV absorbers, generally synthetic and consisting in benzophenones; singlet oxygen quenchers, like carotenoids and polyphenols; oxygen scavengers, that can be inorganic (metal oxides) or consisting in natural compounds like enzymes or ascorbic acid.

Ulvan [116] is a polysaccharide extracted from algae and thus available from marine sources. Ulvan is a hydrosoluble polysaccharide extracted from algae belonging to the Ulva genus, such as *Ulva rigida*, and it represents the main component of their cell walls. Ulvan is a sulfate polysaccharide (mainly including repetition of disaccharides) formed by different percentages of rhamnose sulfate, xylose, urinic acid, and iduronic acid. The composition and structure of ulvan differ in Ulva species [117]. Ulvan can react in water solution, forming a gel with low viscosity. However, its derivatives show an amphiphilic character, useful for packaging [118]. It has shown significant antioxidant properties so that potentialities in food packaging applications are expected in the future [119,120]. Some authors designed new coating film by using ulvan with other polysaccharides to combine different properties and enhance its application.

Ulvan and cellulose combined the antioxidant property with the thermal stability and good barrier of cellulose, producing a barrier against oxygen, UV, and visible light [121].

Other combinations of ulvan with carrageenan produced a new coating film that can increase the gelling property and help the prevention of oil oxidation of food products [122]. Therefore, ulvan represents a new component in packaging products, offering good solubility, barrier, optical, and mechanical properties.

### 3.5. Protective Agents and/or Biopreservatives for Bread Packaging

#### 3.5.1. Essential Oils

Essential oils (EOs) are complex mixtures of volatile compounds produced and stored by several plant species. Overall, terpenes (mono-, sesqui-, and some di-terpenes), in both their hydrocarbon and oxygenated forms, represent the most abundant chemical class in their composition. However, many other chemical groups can be present, such as phenylpropanoids, apocarotenoids, and other volatile non-terpene derivatives (aldehydes, ketones, alcohols, etc.). Despite consumers generally perceiving them as safe products, due to their natural origin, EOs can have human safety issues, such as acute and chronic toxicity by inhalation, allergic reactions, acute oral and dermal toxicity, skin sensitization, skin and eye irritation, photo-sensitization and -carcinogenesis, neurotoxicity, and reproductive toxicity [123]. However, the risk is dose- and composition-dependent, and, especially concerning their use in foods, based on the work of many international organizations, such as the Food and Drug Administration (FDA), the International Organization of Flavor Industries (IOFI), and the Council of Europe (CoE). Each country has produced safety regulations regarding good practice for the processing of plant material, extraction, chemical and toxicological analyses, and the dosage limits [124]. In the US, the FDA has listed the EOs generally recognized as safe (GRAS) that are permitted for use in food for human consumption [125].

Among their biological properties, the antimicrobial efficacy of many EOs has been known for centuries and has been demonstrated by numerous in vitro studies [126,127,128,129] and their antimicrobial potential has also been demonstrated on foodstuffs [130,131,132,133]. EOs have, thus, gained the attention of the scientific community for their use as active ingredients in the food preservation field; regarding the focus of the present review, their use as active ingredients in the preservation of baked products during storage has been evaluated using different techniques, such as (i) the exposure of the product to EO vapors prior to their packaging; (ii) their incorporation in sachets to be inserted in the packaging; (iii) their incorporation in packaging films of different compositions. According to Suhr and Nielsen (2003), the application method to use depends on the composition of the EO, as larger volatiles, such as thymol and eugenol, work best when directly applied to the medium, while smaller molecules, such as citral and isothiocyanates, are more effective when used in vapor form [134].

The exposure of bread to lemongrass (*Cymbopogon citratus* (DC.) Stapf) EO vapor inhibited mold (*Penicillum expansum*) growth on the treated sample for up to 21 days, without significantly interfering with its aroma [135]. On the other hand, the treatment with vapors of marjoram (*Origanum majorana* L.) and clary sage (*Salvia sclarea* L.) EOs effectively controlled the growth of bread spoilage molds, but their aroma persisted on the product for hours after the removal of the package; the panelists deemed the product flavor and taste as “strange and unacceptable” [136].

The problem of the residual aroma in the final product when using EOs as protective agents in active packaging solutions is also reported when using sachets. Oregano (*Origanum vulgare* L.) EO incorporated into a polymeric resin contained in a sachet used in the packaging of bread slices exhibited antimicrobial activity, most probably due to its content in carvacrol and thymol, towards yeasts an molds, as a function of its concentration; however, its residual aroma in the slices packaged with the highest concentration of EO was reported as unpleasant by the panelists [137].

EOs can also be used as active protective agents by incorporation in packaging films. Nanoemulsions of clove bud (*Syzygium aromaticum* (L.) Merr. & L.M.Perry) and oregano (*Origanum vulgare* L.) EOs added to a methylcellulose film reduced the growth of molds and yeasts in sliced bread for up to 15 days [138]. Gliadin films incorporating cinnamaldehyde, the main constituent of cinnamon (*Cinnamomum verum* J.Presl) EO, were highly effective in delaying fungal growth in sliced bread [139]. Cinnamaldehyde was also incorporated in a cellulose-based film used on bakery products, confirming its antifungal efficacy when used as protective ingredient in active packaging [140]. Cinnamon EO incorporated in microperforated polypropylene was also reported as an active packaging agent able to extend the shelf life of several bakery products for up to 10 days; however, to avoid the flavor alteration due to the EO aroma, the package had to be metalized to further minimize its interaction with the contained product [141]. However, except for Gutiérrez et al. (2009), the above-cited studies about the incorporation of EOs in protective films did not report any panel test analysis of the final product, so modifications of the sensory profile (especially the aroma) of the bread packaged with the proposed solutions cannot be excluded and should be further evaluated.

A combined approach, coupling MAP and the incorporation of EO in active packaging, has been reported as the most efficient solution for shelf life improvement for up to 30 days: Suhr and Nielsen (2005) tested mustard (*Brassica nigra* W.D.J.Koch) EO, whose composition is mainly represented by allyl isothiocyanate; they did not report any panel test analysis, however, the EO concentration needed for wheat bread was higher than that required for rye bread, thus implying possible sensory quality alterations in the final product [142].

There are, thus, several factors to take into account regarding the use of EOs in the development of active packaging solutions, such as (i) the possible residual odor of the EOs on the final product, which can impact consumers’ preference; (ii) the different regulations ongoing in each country regarding the safety of the use of EOs; (iii) the in vitro conditions of use to test the antimicrobial activity of EOs may not be applicable in the development of active packaging (i.e., excessive needed concentrations, the composition of the active material, etc.); (iv) different EOs are active towards different microorganisms, thus the appropriate combination of EOs and active packaging must be chosen based on the specific product to store [143]. Moreover, better performances could be achieved using EOs in conjunction with other packaging techniques (such as MAP, low temperature, high pressure, etc.) and/or encapsulated in specifically designed delivery systems.

#### 3.5.2. Lactic Acid Bacteria and Their Metabolites

As part of the growing interest in the search for natural additives for food preservation, a necessary mention must be made of lactic acid bacteria (LAB) and their metabolites. LAB are Gram-positive, non-spore-forming, and fermentative bacteria able to tolerate low pH values in the environment [144]. They are part of the physiological microflora of the human intestine and are generally considered as safe (GRAS), gaining a keen interest from the food industry as biopreservatives [145]. Upon growth in nutrient media such as De Man, Rogosa, and Sharpe broth (MRSB) and/or in foodstuffs, LAB typically produce a wide array of substances including vitamins, short-chain fatty acids, organic acids (lactic and acetic), exopolysaccharides, hydrogen peroxide, bacteriocins (e.g., reuterin, nisin, pediocin), other bacteriocin-like substances, enzymes, and antimicrobial peptides. Many of these substances are endowed with potent antibacterial and antifungal activity so that the direct application of LAB and/or their metabolites to a variety of food matrices has been extensively investigated in order to prevent microbial growth and prolong shelf life [135,145]. The multitude of bioactive metabolites produced by LAB and other probiotic bacteria has been widely referred to as “postbiotics”, a term that has recently also come into use in the field of the food industry [145]. Nevertheless, the term has lately been revised by the International Scientific Association of Probiotics and Prebiotics (ISAPP) to also include in the definition intact non-viable microorganisms or their structural components (e.g., cell wall, membranes, exopolysaccharides, cell-wall-anchored proteins, pili, etc.) with or without metabolite/end products [146]. Beyond definitions, postbiotics are increasingly considered a valid alternative to living probiotics in food biopreservation, as they are considered safer, especially for vulnerable or pediatric individuals, more stable, and easier to produce and store than living cells [147].

The protective effect of postbiotics in the form of cell-free supernatants (CFSs) from *Lacticaseibacillus plantarum* 4F was evaluated by inoculating them directly on slices of bread intentionally pre-contaminated with spores of *Aspergillus fumigatus* [148]. As compared to untreated samples in which about 40% of the bread surface was covered by *A. fumigatus* after 3 days of incubation at 30 °C, samples treated with CFS remained free of fungal growth for more than 6 weeks. Antifungal activity was retained after heat treatment of CFS at 100 °C, while it decreased in the presence of proteinase K and pepsin, suggesting that at least part of the antifungal effect was due to components with a proteinaceous nature [148]. Similar protective effects of CFS were observed in another study, in which four strains of LAB (L. *fermentum* Te007, *Pediococcus pentosaceus* Te010, *L. pentosus* G004, and L. *paracasi* D5) were investigated for their ability to prevent fungal growth on commercial bread [149]. The addition of LAB supernatants to slices of bread resulted in longer shelf life than controls, highly retarding the growth of the molds *A. niger* and *A. oryzae* at different temperatures of incubation (20, 30, and 40 °C).

Despite these promising examples, the direct addition of postbiotics to foodstuffs may have a major drawback as it may alter food properties such as color, odor, or flavor, which would be uncomfortable for consumers. For instance, CFSs produced in MRSB exhibit a typical brown-yellowish color that stains bread slices, giving them an unpleasant appearance, while the organic acid content may confer an acidic taste [149]. Therefore, postbiotics’ incorporation into suitable polymeric films for food packaging could be a valid alternative, allowing the sensory properties of foods to be maintained. In this respect, active food packaging based on the use of postbiotics can be fabricated via several means [147]. For instance, postbiotics can be applied on the surface of polymeric films through coating or adsorption. Alternatively, single postbiotic products such as bacteriocins or enzymes can be covalently linked to the polymer through a ligand. Finally, direct incorporation of postbiotics into the polymeric matrix or lamination of the postbiotic-loaded active film between two external layers, in order to increase postbiotics’ stability and control their migration, could be other modalities [147].

Postbiotic-based food packaging has been investigated for food categories such as meat and dairy products [147], but to the best of our knowledge, application of such a strategy for specifically developing active bread packages is still very poorly investigated. In this regard, Zheng and coworkers combined CFS from *Lacticaseibacillus paracasei* ZX1231 with bacterial nitrocellulose (BNC) films to produce active packaging with antifungal activities. To this aim, dry BNC films (2 cm^2^) were immersed in CFS solutions with different concentrations for different times to identify the optimal conditions for CFS-BNC film preparation. Soaking time displayed a greater impact on the antifungal activity of the CFS-BNC films than the CFS concentration with an optimum reached at a CFS concentration of 63.07% (*w*/*v*) and a BNC soaking time of 60 min. Optimized films were tested for their mechanical properties, thermal degradation, and antifungal activity as bread packaging against *Penicillium*-*polonicum*-inoculated bread slices. The CFS-BNC films became more stretchable but less strong than control films, acquired a more dense appearance according to SEM analysis, and displayed a better thermal stability at a temperature between 0 and 100 °C. Finally, as to the bread test, the adoption of the CFS-BNC film packaging significantly reduced the log CFU/g of *P. polonicum* from 6.5 log at time 0 down to 4.2 log within 15 days. Overall, the results obtained suggest that postbiotic-based films may be a promising strategy for bread packaging. Nevertheless, a fine balance between the active concentrations of postbiotics and retention of the mechanical properties of the films needs to be found to obtain packaging with adequate characteristics [150].

## 4. Promising Biobased Biopolymeric Matrices for Bread Packaging

Waxed paper, paper, or glazed imitation parchment impregnated with paraffin are the oldest materials adopted for bread packaging [151].

Nowadays, bread is mostly packed in bags made of polyolefin fossil-based films such as low-density polyethylene (LDPE) or polypropylene (PP) [152]. However, the massive consumption of these fossil-based plastics contributes to the depletion of natural resources, littering, and global warming [153]. Thanks to the introduction and implementation of the Single-Use Plastic Directive, an acceleration towards alternatives to traditional plastic materials has occurred.

An emerging trend has been registered worldwide related to the adoption of biopolymers as packaging materials driven by the major benefits (eco-friendly nature, possibility to be biodegradable, non-toxicity, excellent film forming, etc.) over conventional plastics [154].

**Figure 6:**
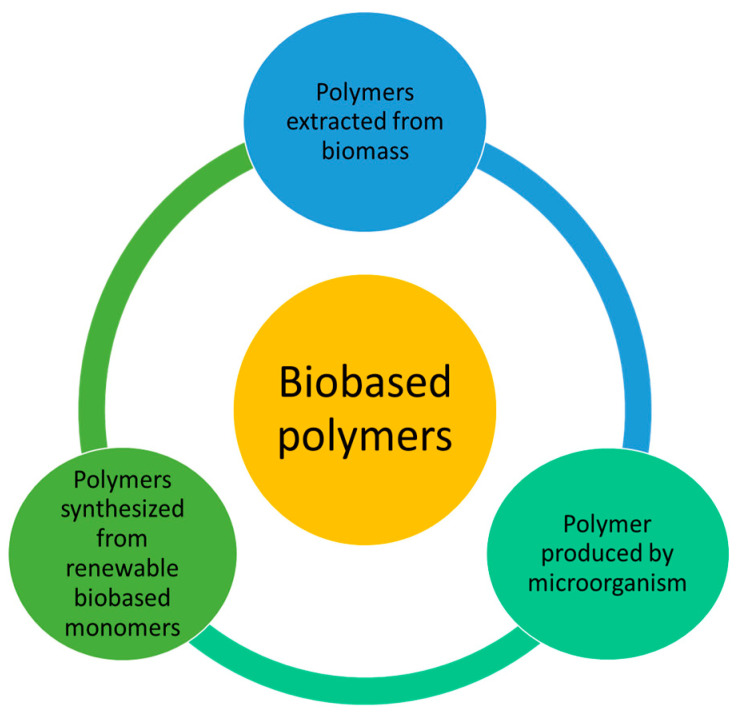
Classification of biobased polymers based on the origin and production process.

Based on their origin and production processes, biobased polymers can be categorized into three main categories (summarized in Figure 6): polymers that are directly extracted from biomass, chemically synthetized polymers starting from renewable biobased monomers, and polymers that are synthetized by microorganisms [155].

In the following sections, the most promising biopolymer matrices for bread-packaging applications are summarized, also evidencing how these matrices can be combined with the biomolecules described in the previous sections.

### 4.1. Starch-Based Materials

Starch is the most abundant form of polysaccharides available in plants. Its renewability and relatively low cost make it very interesting. In the literature, many attempts have been made to develop starch-based plastic. However, to obtain thermoplastic starch, blending with additives is unavoidable due to starch’s lack of stability on water absorption, aged-induced retrogradation, inferior mechanical properties, and poor processability [156]. Starch-based polymer can be produced from potato, corn, wheat, cassava, or tapioca. Generally, thermoplastic starch (TPS) can be obtained from the blending of starch aliphatic polyester, glycerol, and water. A linear aliphatic polyester is added to starch to create compostable films, sheets, plastic bags, etc. [157]. However, it must be pointed out that not all starch-based materials can be adopted for food-packaging applications due to the migration of the additives (generally the plasticizers) adopted [158].

Kechichian et al. [159] tried to use cassava starch films containing antimicrobial cinnamon powder for sliced bread packaging; however, the results achieved were not promising due to the negative effect of bread moisture on the physicochemical properties of the film. On the other hand, encouraging results were obtained by Pankaj [160] who investigated the effect of different grapefruit seed extracts (having antifungal properties) on bionanocomposite films, incorporated with chitosan, based on corn starch. The bionanocomposite films obtained demonstrated low hydrophilicity, high water barrier, and good mechanical properties coupled with a high antifungal activity of stored bread samples at 25 °C, 59% RH for 20 days. Good antimicrobial and antifungal properties to increase the bread shelf life were also obtained by Romanoir et al. [161], who developed antimicrobial starch-based films containing citric acid able to efficiently inhibit the microbial growth in bread samples compared to commercial food wrappers, extending the bread shelf life. Analogous results were obtained by Promhuad et al. [162] who incorporated maltol (that is a widely used flavor enhancer in baked goods and possesses an antimicrobial function) on acetylated cassava starch films. Araùjo et al. [163] developed biodegradable active packaging made of corn starch containing pectin extract and essential oil of turmeric (*Curcuma longa* L.) as a viable solution for sliced bread packaging.

Modified corn starch has also been adopted, as edible coating, for bread preservation by Syafiq et al. [164]; they demonstrated how the edible coating of corn starch, modified with ascorbic acid, added with tomato powder has the potential to extend the shelf life of bread made from frozen dough. Bravin et al. [88] proposed an emulsified edible film composed of corn starch, methylcellulose (MC), and soybean oil to efficiently control moistening in moisture-sensitive bakery products (like crackers).

### 4.2. Cellulose-Based and Lignin-Based Materials

Cellulose is one of the main constituents of paper. However, another important application of cellulose is its use to make cellophane (film). Cellophane film has characteristics of transparency and “dead folds”. Once the film is folded, it is irreversible. One of the major drawbacks in using cellophane is that is not heat-sealable; thus, a sealant layer and/or barrier layer is necessary for its application in packaging [165]. Among the cellulosic derivates, cellulose acetate (CA) has also gained much interest thanks to its excellent biodegradability, excellent optical property, high toughness, low moisture barrier properties, and its capacity to form films [166]. Another interesting cellulose-based material adopted for food packaging is carboxymethyl cellulose (CMC) derived from agriculture waste [167].

Of particular interest is also lignin, that is one of the major readily available renewable resources since it is the second most prevalent biomass component [168]. Thanks to its chemical structure rich in aromatics and phenolic hydroxyls, lignin has good antimicrobial properties, thus being interesting for food-packaging applications [169]. Moreover, lignin possesses good thermal stability and UV barrier properties, and it is non-toxic and biodegradable. Generally, lignin is added as filler to polymeric matrices for obtaining active films for food-packaging applications [170].

Focusing the attention on bread packaging, in the literature there are several examples of cellulose-based and lignin-based films with antimicrobial and antioxidant properties tailored for bread storage and preservation. Some interesting results achieved are summarized in Table 1.

### 4.3. Polylactic-Acid-Based Materials

Polylactic acid is a 100% biobased polymer obtained from the polymerization of lactic acid monomers. Since lactic acid has two stereoisomers, PLA can be found in the form of L-(poly(L-lactide)) or D-(poly(D-lactide) or a combination of both. The D content influences its thermochemical properties [174]. PLA is approved as food contact material, and its compostability in industrial conditions makes it interesting for food-packaging applications. However, to be used for this purpose, the improvement of its barrier, antimicrobial, and thermal properties is necessary [175].

To counterbalance the excessive stiffness of PLA, a softening polymer that also possesses a different viscosity and helps to improve the processability is added. In this context, Suwanamornlert et al. developed an active film made of PLA/PBSA added with thymol as an active molecule [176]. The achieved results are very interesting; in fact, the addition of thymol improved not only the barrier properties but also increased the antifungal films’ properties, achieving an increment of the bread shelf life. Moreover, the presence of thymol reduced the water vapor, oxygen, and carbon dioxide permeability. Thymol was also adopted, coupled with carvacrol, by Lukic et al. [177] in the development of partially biobased biodegradable PLA/PCL films with high antioxidant activity for food-packaging applications. Altan et al. [178] developed a composite fibrous film developed from zein and PLA in which different carvacrol concentrations were added. A sustained diffusion-controlled release behavior was achieved and the preliminary results on bread samples indicated that zein and PLA fibers could be good candidates for bread preservation. Cavalli et al. [179] developed completely biodegradable, but only partially biobased, blends based on: PLA, poly(ethyleneco-vinyl acetate) (EVA), polyethylene glycol (PEG), and chitosan. The presence of chitosan as a natural antifungal agent increased the bread preservation; meanwhile, the EVA balanced the PLA brittleness well and the films obtained possessed very good toughness.

Focusing the attention on PLA-based bread packaging, Table 2 summarizes the main important studies found in the literature.

### 4.4. PHA-Based Materials

PHAs are biodegradable biopolymers obtained from bacterial cells produced as intracellular food and energy reserves for bacteria. PHAs can also be produced by fermentation of renewable sources. Among their homopolymers, poly(3-hydroxybutyrate) (PHB) is the most widespread and best characterized of the PHA family [184]. Studies concerning the use of PHAs for bread packaging are very few in the literature. Sharma et al. [185] developed a biodegradable film of poly(3-hydroxybutyrate-co-4-hydroxybutyrate) containing thyme for prolonging the shelf life of bread. The results obtained are encouraging, the presence of thyme extended the bread shelf life to at least 5 days compared to 1–4 days of the neat film. Mittal et al. [186] developed an active poly(hydroxybutyrate) film incorporated with nanosilica and clove essential oil for packaging of brown bread; the developed films extended the shelf life of brown bread up to 10 days with respect to the bread wrapped in PE.

### 4.5. Other Completely or Partially Biobased Polymeric Matrices

Other completely biobased or partially biobased polymeric matrices synthesized from renewable biobased monomers and used for bread packaging are listed in Table 3.

## 5. Market Challenges of Innovative Biobased/Biodegradable Packaging Solutions for Bread and Bakery Products

### 5.1. Current Status of Packaging Solutions for Bread and Barriers to Overcome for Biobased/Biosegradable Solutions

As documented in the literature, among the various techniques explored in previous sections, the most prevalent bread-packaging system, which has not only remained a subject of research but has also made its way into the market, is modified atmosphere packaging (MAP) [155].

Currently, the most used materials for bread packaging are polyamide (PA), polypropylene (PP), low-density polyethylene (LDPE), and high-density polyethylene (HDPE) [191]. More specifically, the most popular bread-packaging material is glazed imitation parchment (GIP) impregnated on both sides with paraffin wax containing low-density polyethylene (PE-LD) and other petrol-based additives [192]. These solutions provide substantial shelf life to the product but lack sustainability. Despite stricter regulations and a growing demand for sustainability, biobased and biodegradable solutions remain a small percentage within this domain.

Why is it that all the biobased and sustainable solutions described in the previous sections of this review have not gained significant traction on the market despite the increasing emphasis on sustainability and environmental consciousness? The reasons can be summarized in the concept that encouraging sustainable consumer behavior and establishing a business case for adopting more sustainable packaging require a thorough understanding of current consumer driver rules [193]. Moreover, very often, these sustainable solutions are not economically viable or profitable for the industries [194].

Comprehensive assessments and research focusing on consumer dynamics associated with sustainable packaging are necessary, considering the complexities of food-packaging alternatives, increasing regulations, and the need for shared value with consumers. Such consumer research and sustainable packaging decision making are vital for stakeholders in the food and packaging industry [195].

In some cases, marketed innovations claiming to be sustainable through biobased resources or biodegradability often lack a comprehensive environmental assessment. Many of these eco-friendly solutions fall short of expectations, with uncertain renewable resource utilization and compostability claims. Moreover, the key aspect of sustainable food consumption, reducing food loss, is not adequately addressed in these innovations. Bridging the gap between sustainable food consumption and innovative packaging technologies is crucial for enhancing food sustainability [196].

While biopackaging solutions using renewable and biodegradable materials have received attention, technical challenges hinder their widespread adoption among packaging producers [197]. Additionally, the lack of tools to tailor packaging to specific food requirements and accurately assess the sustainability of biopackaging innovations limits their full potential. Addressing these challenges and promoting research and development efforts can unlock promising opportunities for sustainable food-packaging practices.

### 5.2. Examples of Biobased Innovative Solutions on the Market for Bread and Bakery Products

Despite the concerns expressed in the previous paragraph, the market for biobased polymers is expected to achieve a valuation of EUR 30 billion by 2027, displaying a robust CAGR of 14% from 2022 to 2027 [198]. Currently, bioplastics represent less than 1% of the more than 367 Mtons of plastic produced annually; but it has continuously grown thanks to the rising demand combined with the necessity of more sophisticated and sustainable applications and products in relation to environmental concerns [199]. In the previous sections, we discussed the primary polymeric matrices explored in the literature, along with an extensive review of active biomolecules aimed at enhancing specific properties of packaging.

**Figure 7:**
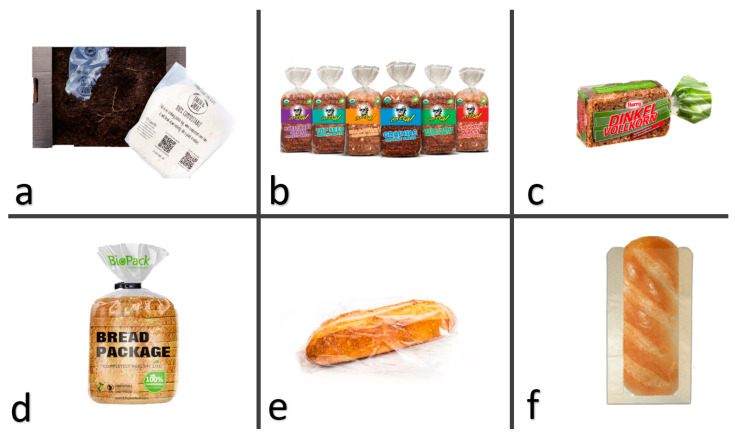
Examples of biobased market solutions for bread packaging: (**a**) C-Bag (**b**) Eureka Bag (**c**) Amerplast bag (**d**) BioPack sealad bag (**e**) BioPack baking bread loaf bag (**f**) ThePureOption bag.

In this subsection, the solutions that have progressed beyond the laboratory scale and have been tested on a pilot scale and put into the market are shown in Figure 7 and detailed as follows.

Earth & Wheat, a D2C company, has made a significant change in its packaging approach by transitioning from a fossil-based bag to a compostable one derived from renewable resources. This eco-friendly packaging (Figure 7a) shows similar properties to LDPE, including impact, puncture, and water resistance. It is now utilized to ship a diverse assortment of bakery bread varieties. The trade name is “C-Bag”, it is made from renewable resources compounding starch and other biopolymers, and it is certified by TUV as OK Home Compostable [200].

Eureka! Baking Co. (Figure 7b) has recently introduced into the market, working closely with supplier Braskem, biobased bread packaging using bio polyethylene, which derives from sugarcane ethanol—a 100% renewable raw material. The sugarcane used is non-GMO, ensuring a natural and environmentally friendly source. Remarkably, for every ton of green plastic produced, approximately 2.15 tons of CO_2_ are sequestered from the atmosphere, contributing to a greener and more eco-conscious packaging solution [201].

Renewable biobased polyethylene is also the raw material used by Amerplast to produce a full range of bakery packaging including films, laminates, and ready-made bags [202] (an example is shown in Figure 7c).

PLA-based plastics are used to make transparent and side-sealed bags produced by BioPack (Figure 7d). They have a thickness of 40 microns, and they are also certified compostable according to European Standard 13432 [203]. BioPack also produces 100% biobased home-compostable cellophane baking bread loaf bags (Figure 7e, [204]).

A mixture of cellulose/PLA has been optimized by ThePureOption to develop biodegradable compostable bakery bread bags made using sustainable cellulose board from well-managed wood stocks and corn-based PLA; this product is also certified home compostable (Figure 7f, [205]).

### 5.3. Material Savings for Improving Bread Shelf life through Personalized Design and/or Processing Optimization

It is noteworthy to underline that, to reduce the material environmental footprint, customized design of packaging applications [206], accompanied by an optimization of the production process parameters [207], will improve the efficiency of the biobased packaging itself and, consequently, the shelf life of the food; indeed, materials innovations have to be accompanied by new packaging designs for representing real opportunities to reduce food waste within the supply chain [208].

Within the realm of designing food packaging, including that of bread, the augmentation of product value is achieved through customization to align with consumer preferences. Personalization is intricately connected to adaptation, allowing for enhancements in packaging properties from various perspectives, encompassing functionality, aesthetics, economics, and ecology [209]. Hence, the meticulous choice of the food-packaging-manufacturing process holds significant importance, with the thermoforming process standing out as one of the most frequently employed methods in this scenario [210].

It is crucial for innovative packaging designs to be accessible for biobased/biodegradable materials suitable for bread. In this context, an effective design has the potential to enhance sustainability by reducing both food and packaging waste at the end of their lifecycle [211]. In this context, ensuring the chemical safety of materials can be advanced through a bottom-up approach using green chemistry principles in the creation of novel biobased and biodegradable materials [212].

Research on the ecotoxicity of biobased materials is actively pursued, particularly post-composting or soil degradation, consistently yielding encouraging findings [213]. Additionally, in contrast to non-biodegradable fossil-based plastics, biodegradable bioplastics have the potential to break down into smaller particles, contributing to microplastic pollution and adversely impacting various plant and animal species [214].

Moving on to practical examples, in the design of multilayer film packaging for bread, various layers can be intricately arranged [215]. To enhance the techno-functional properties of biobased polymers, an internal barrier layer film typically comprises polymers with superior oxygen barrier properties, while polymers exhibiting heightened water vapor barrier properties and mechanical resistance function as the outer layer [216]. The barrier layer comes into direct contact with the external environment, serving as a shield against elements that can lead to the degradation of packaged food, including moisture, oxygen, and microorganisms [217].

Alias et al. [218] have reported that multilayered films incorporating biomass and synthetic biodegradable polymers derived from natural monomers demonstrated superior transparency, water solubility, and mechanical properties compared to single-layer films. Furthermore, achieving high-performance biodegradable multilayer films with customized properties necessitates an understanding of their microstructure and the various steps involved in film processing; the challenge lies in enhancing the protective barrier of individual layers and optimizing matrix design, prompting the redirection of current research efforts in this direction.

Additionally, innovative approaches have emerged for creating micro- and nanosystems responsible for delivering antioxidant and antimicrobial agents in active packaging. However, advancements are still needed in both the design and large-scale production of these active biomaterials for application in the food industry [219]. Cutting-edge research is underway to develop composite materials (also for bread packaging) that incorporate both natural and synthetic additives, employing emerging technologies like 3D printing that has the potential to enhance the functionality of the resulting biocomposites [220].

### 5.4. Future Perspectives and Legislation for the Application of Biobased Materials in Bread-Packaging Fields

As far as our knowledge goes, the applications described in the previous sections still appear to be niche. For bread packaging to truly become sustainable, it is essential to upscale all the biobased solutions that, due to the reasons outlined in Section 5.1, have not yet found their way into the market and remain confined to laboratories.

It is important that the future of packaging for fresh bread could involve a combination of sustainable materials, advanced technology for freshness monitoring, and packaging solutions tailored to consumer preferences and environmental considerations. It is also important for stakeholders in the industry to stay adaptable and responsive to evolving consumer demands and global sustainability goals [221].

Ensuring that the innovative ideas bridging the gap between academic research and market application do not fall into the abyss of neglect is imperative. For sustainable packaging solutions for bread, it is essential that the production and manufacturing processes align with both economic sustainability for companies and international legislation favoring biobased or otherwise sustainable options in bread-packaging applications. These solutions must not only showcase technological prowess but also prove economically viable for both producers and consumers.

Currently, in Europe, a major challenge lies in utilizing natural antimicrobial agents or other biobased additives in bread packaging that are not yet present on official lists for their use. The effectiveness of these agents is described in the literature but the possibility of migration and/or contamination depends on factors like the polymeric matrix and employed technology [191]. Global standards for food packaging are currently undefined, creating significant variations. To ensure future food safety and security, it is essential to integrate diverse technological advancements in novel food packaging. This integration requires a comprehensive examination of food–package interactions, considering legislative concerns. Consequently, conducting a risk-based study on various innovative food-packaging strategies across the food chain becomes critical for assessing and quantifying potential risks [222].

## 6. Conclusions

This review represents the culmination of interdisciplinary efforts, drawing upon the expertise of materials scientists, agricultural scientists, biologists, and experts from various fields to understand and to update the state of the art of the innovative biobased polymeric systems for bread packaging to extend its shelf life.

The integration of active biomolecules within bioplastic matrices represents a promising avenue for enhancing the shelf life of bread products. By improving oxygen and water vapor barrier properties, as well as imparting antimicrobial and antioxidant attributes, this innovative packaging approach holds great potential for addressing the challenges associated with bread spoilage and quality degradation. It is evident that such advancements will play a crucial role in extending the freshness and safety of bread while aligning with sustainability goals through the use of active biomolecules and bioplastics in a synergistic way.

Further research and development efforts in this area are warranted to unlock the full spectrum of benefits and commercial viability of active bioplastic packaging for bread and other perishable goods in order to spread their adoption among packaging producers.

In conclusion, the key to the development and the introduction in the market of innovative biobased materials lies in striking a delicate balance where technological performance meets economic sustainability, fostering a harmonious relationship between producers and consumers in the pursuit of sustainable packaging solutions for bread.

## Figures and Tables

**Table 1:** Main results achieved on bread packaging with cellulose-based and lignin-based materials.

Matrix	Additives and/or Active Compounds Added	Bread-Packaging Improvements Registered	Ref.
**CMC**	Chitosan/zinc oxide nanoparticles	The antimicrobial properties of chitosan–CMC films were tested, and the shelf life of sliced bread increased from 3 to 35 days.	[171]
**CA**	Sodium propionate	Reduced bread mold growth of bread slices.	[172]
**Methylcellulose**	Clove oil and oregano nanoemulsions	Inhibition of yeasts and molds in sliced bread stored for 15 days.	[138]
**Cellulose-derived polymer**	Cinnamaldehyde	Protection of the bread against aerobic mesophiles, yeast, and mold.	[140]
**CA**	Natural additives and oregano essential oil	Improved antifungal properties of hamburger buns.	[173]
**PVA**	Lignin	Inhibition of mold growth on bread samples after 3rd day.	[105]

**Table 2:** Main results achieved on bread packaging with PLA-based materials.

PLA-Based Matrix	Additives and/or Active Compounds Added	Bread-Packaging Improvements Registered	Ref.
**PLA**	Chitosan	Improved capacity to inhibit *S. aureus* on sliced bread. Moreover, the oxygen permeability and the film elongation at break were also improved.	[180]
**PLA/PBAT**	Trans-cinnamaldehyde	Inhibition of mold and yeast growth in sliced bread without altering the sensorial bread properties.	[181]
**PLA/PBSA**	Thymol	Inhibition of fungal growth for bread up to 9 days. Moreover, an improvement of thermal and barrier properties of the film was achieved.	[176]
**PLA/PBAT**	Carvacrol	The PLA/PBAT ratio was optimized to control the films’ strength, permeability, and release behavior of carvacrol. A delay in the fungal growth and sporulation of *Penicillium* sp. and *Rhizopus* on the bread was also achieved.	[182]
**PLA**	Schiff	Delayed growth of fungi on bread slices to day 5 compared with the control system.	[183]

**Table 3:** Main results achieved on bread packaging with biobased polymers such as PBS, PBAT, and PCL.

Matrix	Additives and/or Active Compounds Added	Bread-Packaging Improvements Registered	Ref.
**PBAT/PBS**	-	The formation of oriented fibrous networks subsequently controlled mechanical and barrier properties of the blend films. Blending PBAT and PBS modified the morphology and permeability of biobased films and increased the shelf life of packaged bread.	[187]
**PBS**	Geraniol essential oil	Shelf-life extension study informed that the spoilage of bread stored with an antimicrobial sachet was delayed by more than three weeks.	[188]
**PCL**	Grapefruit seed extract/chitosan	Inhibition of the mold growth on packaged bread with film containing >1.0 mL/g grapefruit seed extract after 7 days.	[189]
**Cashew-gum-gelatin film**	Carvacrol	Extension of the bread shelf life to 6 days with respect to the 3 days of the control system.	[190]

## Data Availability

Data available on request.

## References

[1] Nicolosi A., Laganà V.R., Di Gregorio D. (2023). Habits, Health and Environment in the Purchase of Bakery Products: Consumption Preferences and Sustainable Inclinations before and during COVID-19. Foods.

[2] Apicella A., Scarfato P., Di Maio L., Incarnato L. (2019). Sustainable Active PET Films by Functionalization with Antimicrobial Bio-Coatings. Front. Mater..

[3] Bianchi A., Venturi F., Zinnai A., Taglieri I., Najar B., Macaluso M., Merlani G., Angelini L.G., Tavarini S., Clemente C. (2023). Valorization of an Old Variety of Triticum Aestivum: A Study of Its Suitability for Breadmaking Focusing on Sensory and Nutritional Quality. Foods.

[4] Estévez M., Li Z., Soladoye O.P., Van-Hecke T., Toldrá F. (2017). Health Risks of Food Oxidation. Advances in Food and Nutrition Research.

[5] Pascall M.A., DeAngelo K., Richards J., Arensberg M.B. (2022). Role and Importance of Functional Food Packaging in Specialized Products for Vulnerable Populations: Implications for Innovation and Policy Development for Sustainability. Foods.

[6] Sovacool B.K., Bazilian M., Griffiths S., Kim J., Foley A., Rooney D. (2021). Decarbonizing the Food and Beverages Industry: A Critical and Systematic Review of Developments, Sociotechnical Systems and Policy Options. Renew. Sustain. Energy Rev..

[7] Beghetto V., Gatto V., Samiolo R., Scolaro C., Brahimi S., Facchin M., Visco A. (2023). Plastics Today: Key Challenges and EU Strategies towards Carbon Neutrality: A Review. Environ. Pollut..

[8] Clodoveo M.L., Muraglia M., Fino V., Curci F., Fracchiolla G., Corbo F.F.R. (2021). Overview on Innovative Packaging Methods Aimed to Increase the Shelf-Life of Cook-Chill Foods. Foods.

[9] Meng W., Sun H., Su G. (2023). Plastic Packaging-Associated Chemicals and Their Hazards—An Overview of Reviews. Chemosphere.

[10] Stark N.M., Matuana L.M. (2021). Trends in Sustainable Biobased Packaging Materials: A Mini Review. Mater. Today Sustain..

[11] Rahman M., Islam R., Hasan S., Zzaman W., Rana M.R., Ahmed S., Roy M., Sayem A., Matin A., Raposo A. (2022). A Comprehensive Review on Bio-Preservation of Bread: An Approach to Adopt Wholesome Strategies. Foods.

[12] San H., Laorenza Y., Behzadfar E., Sonchaeng U., Wadaugsorn K., Sodsai J., Kaewpetch T., Promhuad K., Srisa A., Wongphan P. (2022). Functional Polymer and Packaging Technology for Bakery Products. Polymer.

[13] Dong Y., Karboune S. (2021). A Review of Bread Qualities and Current Strategies for Bread Bioprotection: Flavor, Sensory, Rheological, and Textural Attributes. Compr. Rev. Food Sci. Food Saf..

[14] Fraś A., Gołębiewska K., Gołębiewski D., Mańkowski D.R., Boros D., Szecówka P. (2016). Variability in the Chemical Composition of Triticale Grain, Flour and Bread. J. Cereal Sci..

[15] Cauvain S.P., Subramaniam P. (2016). Bread and Other Bakery Products. The Stability and Shelf Life of Food.

[16] Monteiro J.S., Farage P., Zandonadi R.P., Botelho R.B.A., de Oliveira L.d.L., Raposo A., Shakeel F., Alshehri S., Mahdi W.A., Araújo W.M.C. (2021). A Systematic Review on Gluten-Free Bread Formulations Using Specific Volume as a Quality Indicator. Foods.

[17] Barbarisi C., De Vito V., Pellicano M.P., Boscaino F., Balsamo S., Laurino C., Sorrentino G., Volpe M.G. (2019). Bread Chemical and Nutritional Characteristics as Influenced by Food Grade Sea Water. Int. J. Food Prop..

[18] Giannou V., Kessoglou V., Tzia C. (2003). Quality and Safety Characteristics of Bread Made from Frozen Dough. Trends Food Sci. Technol..

[19] Cauvain S., Young L. (2006). Current Approaches to the Classification of Bakery Products. Baked Products.

[20] Cauvain S., Cauvain S.P. (2012). Breadmaking: An Overview. Breadmaking.

[21] Melini V., Melini F. (2018). Strategies to Extend Bread and GF Bread Shelf-Life: From Sourdough to Antimicrobial Active Packaging and Nanotechnology. Fermentation.

[22] Degirmencioglu N., Göcmen D., Inkaya A.N., Aydin E., Guldas M., Gonenc S. (2011). Influence of Modified Atmosphere Packaging and Potassium Sorbate on Microbiological Characteristics of Sliced Bread. J. Food Sci. Technol..

[23] Axel C., Zannini E., Arendt E.K. (2017). Mold Spoilage of Bread and Its Biopreservation: A Review of Current Strategies for Bread Shelf Life Extension. Crit. Rev. Food Sci. Nutr..

[24] Bhise S., Kaur A. (2014). Baking Quality, Sensory Properties and Shelf Life of Bread with Polyols. J. Food Sci. Technol..

[25] Alpers T., Kerpes R., Frioli M., Nobis A., Hoi K.I., Bach A., Jekle M., Becker T. (2021). Impact of Storing Condition on Staling and Microbial Spoilage Behavior of Bread and Their Contribution to Prevent Food Waste. Foods.

[26] Caleb O.J., Mahajan P.V., Al-Said F.A.-J., Opara U.L. (2013). Modified Atmosphere Packaging Technology of Fresh and Fresh-Cut Produce and the Microbial Consequences—A Review. Food Bioprocess Technol..

[27] Van Long N.N., Dantigny P., Barros-Velázquez J. (2016). Fungal Contamination in Packaged Foods. Antimicrobial Food Packaging.

[28] Thirupathi Vasuki M., Kadirvel V., Pejavara Narayana G. (2023). Smart Packaging—An Overview of Concepts and Applications in Various Food Industries. Food Bioeng..

[29] Gigante V., Panariello L., Coltelli M.-B., Danti S., Obisesan K.A., Hadrich A., Staebler A., Chierici S., Canesi I., Lazzeri A. (2021). Liquid and Solid Functional Bio-Based Coatings. Polymer.

[30] Chawla R., Sivakumar S., Kaur H. (2021). Antimicrobial Edible Films in Food Packaging: Current Scenario and Recent Nanotechnological Advancements—A Review. Carbohydr. Polym. Technol. Appl..

[31] Salgado P.R., Di Giorgio L., Musso Y.S., Mauri A.N. (2021). Recent Developments in Smart Food Packaging Focused on Biobased and Biodegradable Polymers. Front. Sustain. Food Syst..

[32] Yildirim S., Röcker B., Pettersen M.K., Nilsen-Nygaard J., Ayhan Z., Rutkaite R., Radusin T., Suminska P., Marcos B., Coma V. (2018). Active Packaging Applications for Food. Compr. Rev. Food Sci. Food Saf..

[33] Han J.-W., Ruiz-Garcia L., Qian J.-P., Yang X.-T. (2018). Food Packaging: A Comprehensive Review and Future Trends. Compr. Rev. Food Sci. Food Saf..

[34] Pico J., Bernal J., Gómez M. (2015). Wheat Bread Aroma Compounds in Crumb and Crust: A Review. Food Res. Int..

[35] Baardseth P., Kvaal K., Lea P., Ellekjær M.R., Færgestad E.M. (2000). The Effects of Bread Making Process and Wheat Quality on French Baguettes. J. Cereal Sci..

[36] Martínez-Anaya M.A. (1996). Enzymes and Bread Flavor. J. Agric. Food Chem..

[37] Cho I.H., Peterson D.G. (2010). Chemistry of Bread Aroma: A Review. Food Sci. Biotechnol..

[38] Schieberle P., Grosch W. (1991). Potent Odorants of the Wheat Bread Crumb Differences to the Crust and Effect of a Longer Dough Fermentation. Z. Lebensm. Unters. Forsch..

[39] Schieberle P., Grosch W. (1994). Potent Odorants of Rye Bread Crust-Differences from the Crumb and from Wheat Bread Crust. Z. Lebensm. Unters. Forsch..

[40] Pozo-Bayón M.A., Guichard E., Cayot N. (2006). Flavor Control in Baked Cereal Products. Food Rev. Int..

[41] Zhou W., Hui Y.H., De Leyn I., Pagani M.A., Rosell C.M., Selman J.D., Therdthai N. (2014). Bakery Products Science and Technology.

[42] Prost C., Poinot P., Rannou C., Arvisenet G. (2012). Bread Aroma. Breadmaking.

[43] Kotsianis I.S., Giannou V., Tzia C. (2002). Production and Packaging of Bakery Products Using MAP Technology. Trends Food Sci. Technol..

[44] Ju J., Xie Y., Yu H., Guo Y., Cheng Y., Zhang R., Yao W. (2020). Synergistic Inhibition Effect of Citral and Eugenol against Aspergillus Niger and Their Application in Bread Preservation. Food Chem..

[45] Seiler D.A.L. (1998). Bakery Products. Principles and Applications of Modified Atmosphere Packaging of Foods.

[46] Qian M., Liu D., Zhang X., Yin Z., Ismail B.B., Ye X., Guo M. (2021). A Review of Active Packaging in Bakery Products: Applications and Future Trends. Trends Food Sci. Technol..

[47] Malathy A.S., Periyar S.S., Subramaniyan V., Subramanian S., Sathiavelu M. (2022). Bread Packaging Techniques and Trends. Ital. J. Food Saf..

[48] Garcia M.V., Copetti M. (2019). Alternative methods for mould spoilage control in bread and bakery products. Int. Food Res. J..

[49] Li H., Wang H., D’Aoust J.-Y., Maurer J. (2012). Salmonella Species. Food Microbiology.

[50] Hernández A., Pérez-Nevado F., Ruiz-Moyano S., Serradilla M.J., Villalobos M.C., Martín A., Córdoba M.G. (2018). Spoilage Yeasts: What Are the Sources of Contamination of Foods and Beverages?. Int. J. Food Microbiol..

[51] Šmídová Z., Rysová J. (2022). Gluten-Free Bread and Bakery Products Technology. Foods.

[52] Lorenzo J.M., Munekata P.E., Dominguez R., Pateiro M., Saraiva J.A., Franco D., Barba F.J., Sant’Ana A.S., Orlien V., Koubaa M. (2018). Chapter 3—Main Groups of Microorganisms of Relevance for Food Safety and Stability: General Aspects and Overall Description. Innovative Technologies for Food Preservation.

[53] Santos J.L.P., Chaves R.D., Sant’Ana A.S. (2017). Estimation of Growth Parameters of Six Different Fungal Species for Selection of Strains to Be Used in Challenge Tests of Bakery Products. Food Biosci..

[54] Licciardello F., Giannone V., Del Nobile M.A., Muratore G., Summo C., Giarnetti M., Caponio F., Paradiso V.M., Pasqualone A. (2017). Shelf Life Assessment of Industrial Durum Wheat Bread as a Function of Packaging System. Food Chem..

[55] Grillo O., Rizzo V., Saccone R., Fallico B., Mazzaglia A., Venora G., Muratore G. (2014). Use of Image Analysis to Evaluate the Shelf Life of Bakery Products. Food Res. Int..

[56] Abdelhamid A.G., El-Dougdoug N.K. (2020). Controlling Foodborne Pathogens with Natural Antimicrobials by Biological Control and Antivirulence Strategies. Heliyon.

[57] Bitrus J., Amadi O.C., Nwagu T.N., Nnamchi C.I., Moneke A.N. (2020). Application of Wild Yeast Isolates from Palm Wine and Honey in Baking of Cassava/Wheat Composite Bread. Food Nutr. Sci..

[58] Saladino F., Quiles J.M., Luciano F.B., Mañes J., Fernández-Franzón M., Meca G. (2017). Shelf Life Improvement of the Loaf Bread Using Allyl, Phenyl and Benzyl Isothiocyanates against Aspergillus Parasiticus. LWT.

[59] Lafuente C., Calpe J., Musto L., Nazareth T.d.M., Dopazo V., Meca G., Luz C. (2023). Preparation of Sourdoughs Fermented with Isolated Lactic Acid Bacteria and Characterization of Their Antifungal Properties. Foods.

[60] Heras-Mozos R., Muriel-Galet V., López-Carballo G., Catalá R., Hernández-Muñoz P., Gavara R. (2018). Active EVOH/PE Bag for Sliced Pan Loaf Based on Garlic as Antifungal Agent and Bread Aroma as Aroma Corrector. Food Packag. Shelf Life.

[61] Bhaskar R., Zo S.M., Narayanan K.B., Purohit S.D., Gupta M.K., Han S.S. (2023). Recent Development of Protein-Based Biopolymers in Food Packaging Applications: A Review. Polym. Test..

[62] Coltelli M.-B., Wild F., Bugnicourt E., Cinelli P., Lindner M., Schmid M., Weckel V., Müller K., Rodriguez P., Staebler A. (2015). State of the Art in the Development and Properties of Protein-Based Films and Coatings and Their Applicability to Cellulose Based Products: An Extensive Review. Coatings.

[63] Farris S., Cozzolino C.A., Introzzi L., Piergiovanni L. (2010). Development and Characterization of a Gelatin-Based Coating with Unique Sealing Properties. J. Appl. Polym. Sci..

[64] Gennadios A., Brandenburg A.H., Park J.W., Weller C.L., Testin R.F. (1994). Water Vapor Permeability of Wheat Gluten and Soy Protein Isolate Films. Ind. Crops Prod..

[65] Tang C.-H., Xiao M.-L., Chen Z., Yang X.-Q. (2011). Influence of Succinylation on the Properties of Cast Films from Red Bean Protein Isolate at Various Plasticizer Levels. J. Appl. Polym. Sci..

[66] Farris S., Introzzi L., Piergiovanni L. (2009). Evaluation of a Bio-Coating as a Solution to Improve Barrier, Friction and Optical Properties of Plastic Films. Packag. Technol. Sci..

[67] Wu F., Misra M., Mohanty A.K. (2021). Challenges and New Opportunities on Barrier Performance of Biodegradable Polymers for Sustainable Packaging. Prog. Polym. Sci..

[68] Tihminlioglu F., Atik İ.D., Özen B. (2010). Water Vapor and Oxygen-Barrier Performance of Corn–Zein Coated Polypropylene Films. J. Food Eng..

[69] Zhang H., Mittal G. (2010). Biodegradable Protein-Based Films from Plant Resources: A Review. Environ. Prog. Sustain. Energy.

[70] Cazón P., Velazquez G., Ramírez J.A., Vázquez M. (2017). Polysaccharide-Based Films and Coatings for Food Packaging: A Review. Food Hydrocoll..

[71] Cherian R.M., Tharayil A., Varghese R.T., Antony T., Kargarzadeh H., Chirayil C.J., Thomas S. (2022). A Review on the Emerging Applications of Nano-Cellulose as Advanced Coatings. Carbohydr. Polym..

[72] Herrera M.A., Mathew A.P., Oksman K. (2017). Barrier and Mechanical Properties of Plasticized and Cross-Linked Nanocellulose Coatings for Paper Packaging Applications. Cellulose.

[73] Szymańska-Chargot M., Chylińska M., Pieczywek P.M., Walkiewicz A., Pertile G., Frąc M., Cieślak K.J., Zdunek A. (2020). Evaluation of Nanocomposite Made of Polylactic Acid and Nanocellulose from Carrot Pomace Modified with Silver Nanoparticles. Polymer.

[74] Guivier M., Almeida G., Domenek S., Chevigny C. (2023). Resilient High Oxygen Barrier Multilayer Films of Nanocellulose and Polylactide. Carbohydr. Polym..

[75] Coltelli M.-B., Panariello L., Vannozzi A., Gigante V., Gagliardini A., Morganti P., Cinelli P., Lazzeri A., De Bonise A., Falabella P. (2022). Chitin and Its Derivatives: Nanostructured Materials from Different Marine and Terrestrial Sources. Chem. Eng. Trans..

[76] Coltelli M., Aliotta L., Vannozzi A., Morganti P., Fusco A., Donnarumma G., Lazzeri A. (2020). Properties and Skin Compatibility of Films Based on Poly (Lactic Acid) (PLA) Bionanocomposites Incorporating Chitin Nanofibrils (CN). J. Funct. Biomater..

[77] Hahn T., Tafi E., Paul A., Salvia R., Falabella P., Zibek S. (2020). Current State of Chitin Purification and Chitosan Production from Insects. J. Chem. Technol. Biotechnol..

[78] Kopacic S., Walzl A., Zankel A., Leitner E., Bauer W. (2018). Alginate and Chitosan as a Functional Barrier for Paper-Based Packaging Materials. Coatings.

[79] Priyadarshi R., Rhim J.-W. (2020). Chitosan-Based Biodegradable Functional Films for Food Packaging Applications. Innov. Food Sci. Emerg. Technol..

[80] Bhardwaj A., Sharma N., Sharma V., Alam T., Sahu J.K., Hamid H. (2022). Assessing the Consumer Acceptance and Storability of Chitosan and Beeswax Coated Cellulose Packaging for Whole Wheat Bread. Food Control.

[81] Ji Y., Waters S., Lim E., Lang A.W., Ciesielski P.N., Shofner M.L., Reynolds J.R., Meredith J.C. (2022). Minimizing Oxygen Permeability in Chitin/Cellulose Nanomaterial Coatings by Tuning Chitin Deacetylation. ACS Sustain. Chem. Eng..

[82] Benbettaïeb N., Kurek M., Bornaz S., Debeaufort F. (2014). Barrier, Structural and Mechanical Properties of Bovine Gelatin–Chitosan Blend Films Related to Biopolymer Interactions. J. Sci. Food Agric..

[83] Trinh B.M., Chang B.P., Mekonnen T.H. (2023). The Barrier Properties of Sustainable Multiphase and Multicomponent Packaging Materials: A Review. Prog. Mater. Sci..

[84] Hirte A., Hamer R.J., Meinders M.B.J., Primo-Martín C. (2010). Permeability of Crust Is Key to Crispness Retention. J. Cereal Sci..

[85] Follain N., Chappey C., Dargent E., Chivrac F., Crétois R., Marais S. (2014). Structure and Barrier Properties of Biodegradable Polyhydroxyalkanoate Films. J. Phys. Chem. C.

[86] Reddy M.M., Mohanty A.K., Misra M. (2012). Biodegradable Blends from Plasticized Soy Meal, Polycaprolactone, and Poly (Butylene Succinate). Macromol. Mater. Eng..

[87] Spotti M.L., Cecchini J.P., Spotti M.J., Carrara C.R. (2016). Brea Gum (from *Cercidium praecox*) as a Structural Support for Emulsion-Based Edible Films. LWT Food Sci. Technol..

[88] Bravin B., Peressini D., Sensidoni A. (2006). Development and Application of Polysaccharide–Lipid Edible Coating to Extend Shelf-Life of Dry Bakery Products. J. Food Eng..

[89] Simões A., Coelhoso I.M., Alves V.D., Brazinha C. (2023). Recovery and Purification of Cutin from Tomato By-Products for Application in Hydrophobic Films. Membranes.

[90] Tedeschi G., Benitez J.J., Ceseracciu L., Dastmalchi K., Itin B., Stark R.E., Heredia A., Athanassiou A., Heredia-Guerrero J.A. (2018). Sustainable Fabrication of Plant Cuticle-Like Packaging Films from Tomato Pomace Agro-Waste, Beeswax, and Alginate. ACS Sustain. Chem. Eng..

[91] Manrich A., Moreira F.K.V., Otoni C.G., Lorevice M.V., Martins M.A., Mattoso L.H.C. (2017). Hydrophobic Edible Films Made up of Tomato Cutin and Pectin. Carbohydr. Polym..

[92] Righetti G.I.C., Nasti R., Beretta G., Levi M., Turri S., Suriano R. (2023). Unveiling the Hidden Properties of Tomato Peels: Cutin Ester Derivatives as Bio-Based Plasticizers for Polylactic Acid. Polymer.

[93] Mousavi Khaneghah A., Hashemi S.M.B., Limbo S. (2018). Antimicrobial Agents and Packaging Systems in Antimicrobial Active Food Packaging: An Overview of Approaches and Interactions. Food Bioprod. Process..

[94] Saranraj P., Sivasakthivelan P., Rosell C.M., Bajerska J., El Sheikha A.F. (2015). Microorganisms Involved in Spoilage of Bread and Its Control Measures. Bread and Its Fortification: Nutrition and Health Benefits.

[95] Thompson J.M., Dodd C.E.R., Waites W.M. (1993). Spoilage of Bread by Bacillus. Int. Biodeterior. Biodegrad..

[96] Nielsen P.V., Rios R. (2000). Inhibition of Fungal Growth on Bread by Volatile Components from Spices and Herbs, and the Possible Application in Active Packaging, with Special Emphasis on Mustard Essential Oil. Int. J. Food Microbiol..

[97] LaCoste A., Schaich K.M., Zumbrunnen D., Yam K.L. (2005). Advancing Controlled Release Packaging through Smart Blending. Packag. Technol. Sci..

[98] Brockgreitens J., Abbas A. (2016). Responsive Food Packaging: Recent Progress and Technological Prospects. Compr. Rev. Food Sci. Food Saf..

[99] Majid I., Ahmad Nayik G., Mohammad Dar S., Nanda V. (2018). Novel Food Packaging Technologies: Innovations and Future Prospective. J. Saudi Soc. Agric. Sci..

[100] Fadiji T., Rashvand M., Daramola M.O., Iwarere S.A. (2023). A Review on Antimicrobial Packaging for Extending the Shelf Life of Food. Processes.

[101] Jideani V.A., Vogt K. (2016). Antimicrobial Packaging for Extending the Shelf Life of Bread—A Review. Crit. Rev. Food Sci. Nutr..

[102] Coban H.B. (2020). Organic Acids as Antimicrobial Food Agents: Applications and Microbial Productions. Bioprocess. Biosyst. Eng..

[103] Singh A.K., Kim J.Y., Lee Y.S. (2022). Phenolic Compounds in Active Packaging and Edible Films/Coatings: Natural Bioactive Molecules and Novel Packaging Ingredients. Molecules.

[104] Coiai S., Campanella B., Paulert R., Cicogna F., Bramanti E., Lazzeri A., Pistelli L., Coltelli M.-B. (2021). Rosmarinic Acid and Ulvan from Terrestrial and Marine Sources in Anti-Microbial Bionanosystems and Biomaterials. Appl. Sci..

[105] Baite T.N., Purkait M.K., Mandal B. (2023). Synthesis of Lignin from Waste Leaves and Its Potential Application for Bread Packaging: A Waste Valorization Approach. Int. J. Biol. Macromol..

[106] Yang W., Fortunati E., Dominici F., Giovanale G., Mazzaglia A., Balestra G.M., Kenny J.M., Puglia D. (2016). Effect of Cellulose and Lignin on Disintegration, Antimicrobial and Antioxidant Properties of PLA Active Films. Int. J. Biol. Macromol..

[107] Sachdeva A., Vashist S., Chopra R., Puri D. (2017). Antimicrobial Activity of Active Packaging Film to Prevent Bread Spoilage. Int. J. Food Sci. Nutr..

[108] Wang L., Zhang Y., Xing Q., Xu J., Li L. (2023). Quality and Microbial Diversity of Homemade Bread Packaged in Cinnamaldehyde Loaded Poly(Lactic Acid)/Konjac Glucomannan/Wheat Gluten Bilayer Film during Storage. Food Chem..

[109] Nionelli L., Wang Y., Pontonio E., Immonen M., Rizzello C.G., Maina H.N., Katina K., Coda R. (2020). Antifungal Effect of Bioprocessed Surplus Bread as Ingredient for Bread-Making: Identification of Active Compounds and Impact on Shelf-Life. Food Control.

[110] Panariello L., Coltelli M.-B., Buchignani M., Lazzeri A. (2019). Chitosan and Nano-Structured Chitin for Biobased Anti-Microbial Treatments onto Cellulose Based Materials. Eur. Polym. J..

[111] Panariello L., Coltelli M.-B., Hadrich A., Braca F., Fiori S., Haviv A., Miketa F., Lazzeri A., Staebler A., Gigante V. (2022). Antimicrobial and Gas Barrier Crustaceans and Fungal Chitin-Based Coatings on Biodegradable Bioplastic Films. Polymer.

[112] Malhotra B., Keshwani A., Kharkwal H. (2015). Antimicrobial Food Packaging: Potential and Pitfalls. Front. Microbiol..

[113] Motelica L., Ficai D., Ficai A., Oprea O.C., Kaya D.A., Andronescu E. (2020). Biodegradable Antimicrobial Food Packaging: Trends and Perspectives. Foods.

[114] Rangaraj V.M., Rambabu K., Banat F., Mittal V. (2021). Natural Antioxidants-Based Edible Active Food Packaging: An Overview of Current Advancements. Food Biosci..

[115] Nanditha B., Prabhasankar P. (2008). Antioxidants in Bakery Products: A Review. Crit. Rev. Food Sci. Nutr..

[116] Cicogna F., Passaglia E., Benedettini M., Oberhauser W., Ishak R., Signori F., Coiai S. (2023). Rosmarinic and Glycyrrhetinic Acid-Modified Layered Double Hydroxides as Functional Additives for Poly(Lactic Acid)/Poly(Butylene Succinate) Blends. Molecules.

[117] Lahaye M., Robic A. (2007). Structure and Functional Properties of Ulvan, a Polysaccharide from Green Seaweeds. Biomacromolecules.

[118] Cindana Mo’o F.R., Wilar G., Devkota H.P., Wathoni N. (2020). Ulvan, a Polysaccharide from Macroalga *Ulva* Sp.: A Review of Chemistry, Biological Activities and Potential for Food and Biomedical Applications. Appl. Sci..

[119] Kidgell J.T., Magnusson M., de Nys R., Glasson C.R.K. (2019). Ulvan: A Systematic Review of Extraction, Composition and Function. Algal Res..

[120] Li C., Tang T., Du Y., Jiang L., Yao Z., Ning L., Zhu B. (2023). Ulvan and Ulva Oligosaccharides: A Systematic Review of Structure, Preparation, Biological Activities and Applications. Bioresour. Bioprocess..

[121] Gomaa M., Al-Badaani A.A., Hifney A.F., Adam M.S. (2022). Utilization of Cellulose and Ulvan from the Green Seaweed *Ulva* Lactuca in the Development of Composite Edible Films with Natural Antioxidant Properties. J. Appl. Phycol..

[122] Ramu Ganesan A., Shanmugam M., Bhat R. (2018). Producing Novel Edible Films from Semi Refined Carrageenan (SRC) and Ulvan Polysaccharides for Potential Food Applications. Int. J. Biol. Macromol..

[123] Burfield T. (2000). Safety of Essential Oils. Int. J. Aromather..

[124] Dima C., Dima S. (2015). Essential Oils in Foods: Extraction, Stabilization, and Toxicity. Curr. Opin. Food Sci..

[125] Food and Drug Administration (2012). Code of Federal Regulations 182.20—Essential Oils, Oleoresins (Solvent-Free), and Natural Extractives (Including Distillates).

[126] Romeo F.V., De Luca S., Piscopo A., Poiana M. (2008). Antimicrobial Effect of Some Essential Oils. J. Essent. Oil Res..

[127] Lv F., Liang H., Yuan Q., Li C. (2011). In Vitro Antimicrobial Effects and Mechanism of Action of Selected Plant Essential Oil Combinations against Four Food-Related Microorganisms. Food Res. Int..

[128] El-Said H., Ashgar S.S., Bader A., AlQathama A., Halwani M., Ascrizzi R., Flamini G. (2021). Essential Oil Analysis and Antimicrobial Evaluation of Three Aromatic Plant Species Growing in Saudi Arabia. Molecules.

[129] Wogiatzi E., Gougoulias N., Papachatzis A., Vagelas I., Chouliaras N. (2009). Chemical Composition and Antimicrobial Effects of Greek Origanum Species Essential Oil. Biotechnol. Biotechnol. Equip..

[130] Ghabraie M., Vu K.D., Tata L., Salmieri S., Lacroix M. (2016). Antimicrobial Effect of Essential Oils in Combinations against Five Bacteria and Their Effect on Sensorial Quality of Ground Meat. LWT Food Sci. Technol..

[131] Leonelli Pires de Campos A.C., Saldanha Nandi R.D., Scandorieiro S., Gonçalves M.C., Reis G.F., Dibo M., Medeiros L.P., Panagio L.A., Fagan E.P., Takayama Kobayashi R.K. (2022). Antimicrobial Effect of *Origanum vulgare* (L.) Essential Oil as an Alternative for Conventional Additives in the Minas Cheese Manufacture. LWT.

[132] Djenane D., Yangüela J., Amrouche T., Boubrit S., Boussad N., Roncalés P. (2011). Chemical Composition and Antimicrobial Effects of Essential Oils of *Eucalyptus globulus*, *Myrtus communis* and *Satureja hortensis* against *Escherichia coli* O157:H7 and Staphylococcus Aureus in Minced Beef. Food Sci. Technol. Int..

[133] Klein G., Rüben C., Upmann M. (2013). Antimicrobial Activity of Essential Oil Components against Potential Food Spoilage Microorganisms. Curr. Microbiol..

[134] Suhr K.I., Nielsen P.V. (2003). Antifungal Activity of Essential Oils Evaluated by Two Different Application Techniques against Rye Bread Spoilage Fungi. J. Appl. Microbiol..

[135] Mani López E., Valle Vargas G.P., Palou E., López Malo A. (2018). *Penicillium expansum* Inhibition on Bread by Lemongrass Essential Oil in Vapor Phase. J. Food Prot..

[136] Krisch J., Rentskenhand T., Horváth G., Vágvölgyi C. (2013). Acta Biologica Szegediensis Activity of Essential Oils in Vapor Phase against Bread Spoilage Fungi. Acta Biol. Szeged..

[137] Passarinho A.T.P., Dias N.F., Camilloto G.P., Cruz R.S., Otoni C.G., Moraes A.R.F., Soares N.D.F.F. (2014). Sliced Bread Preservation through Oregano Essential Oil-Containing Sachet. J. Food Process Eng..

[138] Otoni C.G., Pontes S.F., Medeiros E.A., Soares N.D.F. (2014). Edible Films from Methylcellulose and Nanoemulsions of Clove Bud (*Syzygium aromaticum*) and Oregano (*Origanum vulgare*) Essential Oils as Shelf Life Extenders for Sliced Bread. J. Agric. Food Chem..

[139] Balaguer M.P., Lopez-Carballo G., Catala R., Gavara R., Hernandez-Munoz P. (2013). Antifungal Properties of Gliadin Films Incorporating Cinnamaldehyde and Application in Active Food Packaging of Bread and Cheese Spread Foodstuffs. Int. J. Food Microbiol..

[140] Lopes F.A., De Fátima N., Soares F., De Cássia C., Lopes P., Azevedo Da Silva W., Carlos J., Júnior B., Antonio E., Medeiros A. (2014). Conservation of Bakery Products through Cinnamaldehyde Antimicrobial Films. Packag. Technol. Sci..

[141] Gutiérrez L., Sánchez C., Batlle R., Nerín C. (2009). New Antimicrobial Active Package for Bakery Products. Trends Food Sci. Technol..

[142] Suhr K.I., Nielsen P.V. (2005). Inhibition of Fungal Growth on Wheat and Rye Bread by Modified Atmosphere Packaging and Active Packaging Using Volatile Mustard Essential Oil. J. Food Sci..

[143] Ju J., Chen X., Xie Y., Yu H., Guo Y., Cheng Y., Qian H., Yao W. (2019). Application of Essential Oil as a Sustained Release Preparation in Food Packaging. Trends Food Sci. Technol..

[144] Sharafi H., Divsalar E., Rezaei Z., Liu S.-Q., Moradi M. (2023). The Potential of Postbiotics as a Novel Approach in Food Packaging and Biopreservation: A Systematic Review of the Latest Developments. Crit. Rev. Food Sci. Nutr..

[145] Zapaśnik A., Sokołowska B., Bryła M. (2022). Role of Lactic Acid Bacteria in Food Preservation and Safety. Foods.

[146] Salminen S., Collado M.C., Endo A., Hill C., Lebeer S., Quigley E.M.M., Sanders M.E., Shamir R., Swann J.R., Szajewska H. (2021). The International Scientific Association of Probiotics and Prebiotics (ISAPP) Consensus Statement on the Definition and Scope of Postbiotics. Nat. Rev. Gastroenterol. Hepatol..

[147] Moradi M., Kousheh S.A., Almasi H., Alizadeh A., Guimarães J.T., Yılmaz N., Lotfi A. (2020). Postbiotics Produced by Lactic Acid Bacteria: The next Frontier in Food Safety. Compr. Rev. Food Sci. Food Saf..

[148] El oirdi S., Lakhlifi T., Bahar A.A., Yatim M., Rachid Z., Belhaj A. (2021). Isolation and Identification of Lactobacillus Plantarum 4F, a Strain with High Antifungal Activity, Fungicidal Effect, and Biopreservation Properties of Food. J. Food Process Preserv..

[149] Muhialdin B.J., Hassan Z., Sadon S.K. (2011). Antifungal Activity of *Lactobacillus fermentum* Te007, *Pediococcus pentosaceus* Te010, *Lactobacillus pentosus* G004, and *L. paracasi* D5 on Selected Foods. J. Food Sci..

[150] Zheng X., Nie W., Xu J., Zhang H., Liang X., Chen Z. (2022). Characterization of Antifungal Cyclic Dipeptides of *Lacticaseibacillus paracasei* ZX1231 and Active Packaging Film Prepared with Its Cell-Free Supernatant and Bacterial Nanocellulose. Food Res. Int..

[151] Galić K., Ćurić D., Gabrić D. (2009). Shelf Life of Packaged Bakery Goods—A Review. Crit. Rev. Food Sci. Nutr..

[152] Pasqualone A. (2019). Bread Packaging: Features and Functions. Flour and Breads and Their Fortification in Health and Disease Prevention.

[153] Nilsen-Nygaard J., Fernández E.N., Radusin T., Rotabakk B.T., Sarfraz J., Sharmin N., Sivertsvik M., Sone I., Pettersen M.K. (2021). Current Status of Biobased and Biodegradable Food Packaging Materials: Impact on Food Quality and Effect of Innovative Processing Technologies. Compr. Rev. Food Sci. Food Saf..

[154] Perera K.Y., Jaiswal A.K., Jaiswal S. (2023). Biopolymer-Based Sustainable Food Packaging Materials: Challenges, Solutions, and Applications. Foods.

[155] Reichert C.L., Bugnicourt E., Coltelli M.B., Cinelli P., Lazzeri A., Canesi I., Braca F., Martínez B.M., Alonso R., Agostinis L. (2020). Bio-Based Packaging: Materials, Modifications, Industrial Applications and Sustainability. Polymers.

[156] Vázquez A., Foresti M.L., Cyras V. (2011). Production, Chemistry and Degradation of Starch-Based Polymers. Biopolymers—New Materials for Sustainable Films and Coatings.

[157] Jiang T., Duan Q., Zhu J., Liu H., Yu L. (2020). Starch-Based Biodegradable Materials: Challenges and Opportunities. Adv. Ind. Eng. Polym. Res..

[158] Navasingh R.J.H., Gurunathan M.K., Nikolova M.P., Królczyk J.B. (2023). Sustainable Bioplastics for Food Packaging Produced from Renewable Natural Sources. Polymer.

[159] Kechichian V., Ditchfield C., Veiga-Santos P., Tadini C.C. (2010). Natural Antimicrobial Ingredients Incorporated in Biodegradable Films Based on Cassava Starch. LWT Food Sci. Technol..

[160] Jha P. (2020). Effect of Grapefruit Seed Extract Ratios on Functional Properties of Corn Starch-Chitosan Bionanocomposite Films for Active Packaging. Int. J. Biol. Macromol..

[161] Romainor A.N., Chin S.F., Lihan S. (2022). Antimicrobial Starch-Based Film for Food Packaging Application. Starch Stärke.

[162] Promhuad K., Bumbudsanpharoke N., Wadaugsorn K., Sonchaeng U., Harnkarnsujarit N. (2022). Maltol-Incorporated Acetylated Cassava Starch Films for Shelf-Life-Extension Packaging of Bakery Products. Polymer.

[163] Araújo M.N.P., Grisi C.V.B., Duarte C.R., de Almeida Y.M.B., Vinhas G.M. (2023). Active Packaging of Corn Starch with Pectin Extract and Essential Oil of Turmeric Longa Linn: Preparation, Characterization and Application in Sliced Bread. Int. J. Biol. Macromol..

[164] Syafiq R., Sapuan S.M., Zuhri M.Y.M., Ilyas R.A., Nazrin A., Sherwani S.F.K., Khalina A. (2020). Antimicrobial Activities of Starch-Based Biopolymers and Biocomposites Incorporated with Plant Essential Oils: A Review. Polymer.

[165] Putranda Y. (2017). Impact of Biobased Packaging Materials on Quality of Fully-Baked Frozen Bread. Master Thesis.

[166] Rajeswari A., Christy E.J.S., Swathi E., Pius A. (2020). Fabrication of Improved Cellulose Acetate-Based Biodegradable Films for Food Packaging Applications. Environ. Chem. Ecotoxicol..

[167] Yaradoddi J.S., Banapurmath N.R., Ganachari S.V., Soudagar M.E.M., Mubarak N.M., Hallad S., Hugar S., Fayaz H. (2020). Biodegradable Carboxymethyl Cellulose Based Material for Sustainable Packaging Application. Sci. Rep..

[168] Yang W., Ding H., Puglia D., Kenny J.M., Liu T., Guo J., Wang Q., Ou R., Xu P., Ma P. (2022). Bio-Renewable Polymers Based on Lignin-Derived Phenol Monomers: Synthesis, Applications, and Perspectives. SusMat.

[169] Avella A., Ruda M., Gioia C., Sessini V., Roulin T., Carrick C., Verendel J., Lo Re G. (2023). Lignin Valorization in Thermoplastic Biomaterials: From Reactive Melt Processing to Recyclable and Biodegradable Packaging. Chem. Eng. J..

[170] Botta L., Titone V., Teresi R., Scarlata M.C., Lo Re G., La Mantia F.P., Lopresti F. (2022). Biocomposite PBAT/Lignin Blown Films with Enhanced Photo-Stability. Int. J. Biol. Macromol..

[171] Noshirvani N., Ghanbarzadeh B., Mokarram R.R., Hashemi M. (2017). Novel Active Packaging Based on Carboxymethyl Cellulose-Chitosan-ZnO NPs Nanocomposite for Increasing the Shelf Life of Bread. Food Packag. Shelf Life.

[172] Soares N.F.F., Rutishauser D.M., Melo N., Cruz R.S., Andrade N.J. (2002). Inhibition of Microbial Growth in Bread through Active Packaging. Packag. Technol. Sci..

[173] Fernandes F.G., Grisi C.V.B., Costa Araújo R., Botrel D.A., Sousa S. (2022). Active Cellulose Acetate-oregano Essential Oil Films to Conservation of Hamburger Buns: Antifungal, Analysed Sensorial and Mechanical Properties. Packag. Technol. Sci..

[174] Aliotta L., Cinelli P., Coltelli M.B., Righetti M.C., Gazzano M., Lazzeri A. (2017). Effect of Nucleating Agents on Crystallinity and Properties of Poly (Lactic Acid) (PLA). Eur. Polym. J..

[175] Rojas A., Velásquez E., Vidal C.P., Guarda A., Galotto M.J., de Dicastillo C.L. (2021). Active PLA Packaging Films: Effect of Processing and the Addition of Natural Antimicrobials and Antioxidants on Physical Properties, Release Kinetics, and Compostability. Antioxidants.

[176] Suwanamornlert P., Kerddonfag N., Sane A., Chinsirikul W., Zhou W., Chonhenchob V. (2020). Poly(Lactic Acid)/Poly(Butylene-Succinate-Co-Adipate) (PLA/PBSA) Blend Films Containing Thymol as Alternative to Synthetic Preservatives for Active Packaging of Bread. Food Packag. Shelf Life.

[177] Lukic I., Vulic J., Ivanovic J. (2020). Antioxidant Activity of PLA/PCL Films Loaded with Thymol and/or Carvacrol Using ScCO_2_ for Active Food Packaging. Food Packag. Shelf Life.

[178] Altan A., Aytac Z., Uyar T. (2018). Carvacrol Loaded Electrospun Fibrous Films from Zein and Poly(Lactic Acid) for Active Food Packaging. Food Hydrocoll..

[179] Cavalli L.R., Klein J.M., Sandri I.G., Brandalise R. (2021). Este Trabajo Se Centró En El Desarrollo de Envases Activos Biodegradables Con Mezclas de Poli (Ácido Láctico) (PLA), Poli (Etileno-Co-Acetato de Vinilo) (EVA), Polietilenglicol (PEG) y Quitosano (QUI). Se Investigaron Las Características Morfológicas Térm. Res. Soc. Dev..

[180] Kongkaoroptham P., Piroonpan T., Pasanphan W. (2021). Chitosan Nanoparticles Based on Their Derivatives as Antioxidant and Antibacterial Additives for Active Bioplastic Packaging. Carbohydr. Polym..

[181] Srisa A., Harnkarnsujarit N. (2020). Antifungal Films from Trans-Cinnamaldehyde Incorporated Poly(Lactic Acid) and Poly(Butylene Adipate-Co-Terephthalate) for Bread Packaging. Food Chem..

[182] Klinmalai P., Srisa A., Laorenza Y., Katekhong W., Harnkarnsujarit N. (2021). Antifungal and Plasticization Effects of Carvacrol in Biodegradable Poly (Lactic Acid) and Poly(Butylene Adipate Terephthalate) Blend Films for Bakery Packaging. LWT.

[183] Natesan S., Samuel J.S., Srinivasan A.K. (2022). Design and Development of Schiff’s Base (SB)-Modified Polylactic Acid (PLA) Antimicrobial Film for Packaging Applications. Polym. Bull..

[184] Sudesh K., Abe H. (2010). Practical Guide to Microbial Polyhydroxyalkanoates.

[185] Sharma P., Ahuja A., Dilsad Izrayeel A.M., Samyn P., Rastogi V.K. (2022). Physicochemical and Thermal Characterization of Poly (3-Hydroxybutyrate-Co-4-Hydroxybutyrate) Films Incorporating Thyme Essential Oil for Active Packaging of White Bread. Food Control.

[186] Mittal M., Ahuja S., Yadav A., Aggarwal N.K. (2023). Development of Poly(Hydroxybutyrate) Film Incorporated with Nano Silica and Clove Essential Oil Intended for Active Packaging of Brown Bread. Int. J. Biol. Macromol..

[187] Bumbudsanpharoke N., Wongphan P., Promhuad K., Leelaphiwat P., Harnkarnsujarit N. (2022). Morphology and Permeability of Bio-Based Poly(Butylene Adipate-Co-Terephthalate) (PBAT), Poly(Butylene Succinate) (PBS) and Linear Low-Density Polyethylene (LLDPE) Blend Films Control Shelf-Life of Packaged Bread. Food Control.

[188] Petchwattana N., Naknaen P., Cha-Aim B.K., Suksri C., Sanetuntikul J. (2021). Controlled Release Antimicrobial Sachet Prepared from Poly(Butylene Succinate)/Geraniol and Ethylene Vinyl Alcohol Coated Paper for Bread Shelf-Life Extension Application. Int. J. Biol. Macromol..

[189] Kwee B., Lim H., Thian S. (2022). Effects of Molecular Weight of Chitosan in a Blend with Polycaprolactone and Grapefruit Seed Extract for Active Packaging and Biodegradation. Food Packag. Shelf Life.

[190] Oliveira M.A., Gonzaga M.L.C., Bastos M.S.R., Magalhães H.C.R., Benevides S.D., Furtado R.F., Zambelli R.A., Garruti D.S. (2020). Packaging with Cashew Gum/Gelatin/Essential Oil for Bread: Release Potential of the Citral. Food Packag. Shelf Life.

[191] González-López M.E., Calva-Estrada S.d.J., Gradilla-Hernández M.S., Barajas-Álvarez P. (2023). Current Trends in Biopolymers for Food Packaging: A Review. Front. Sustain. Food Syst..

[192] Martins I.E., Shittu T.A., Onabanjo O.O., Adesina A.D., Soares A.G., Okolie P.I., Kupoluyi A.O., Ojo O.A., Obadina A.O. (2021). Effect of Packaging Materials and Storage Conditions on the Microbial Quality of Pearl Millet Sourdough Bread. J. Food Sci. Technol..

[193] Ghali-Zinoubi Z. (2022). Examining Drivers of Environmentally Conscious Consumer Behavior: Theory of Planned Behavior Extended with Cultural Factors. Sustainability.

[194] Tarazona N.A., Machatschek R., Balcucho J., Castro-Mayorga J.L., Saldarriaga J.F., Lendlein A. (2022). Opportunities and Challenges for Integrating the Development of Sustainable Polymer Materials within an International Circular (Bio)Economy Concept. MRS Energy Sustain..

[195] Morashti J., An Y., Jang H. (2022). A Systematic Literature Review of Sustainable Packaging in Supply Chain Management. Sustainability.

[196] Prieto-Sandoval V., Torres-Guevara L.E., García-Díaz C. (2022). Green Marketing Innovation: Opportunities from an Environmental Education Analysis in Young Consumers. J. Clean. Prod..

[197] Agarwal A., Shaida B., Rastogi M., Singh N.B. (2023). Food Packaging Materials with Special Reference to Biopolymers-Properties and Applications. Chem. Afr..

[198] Joseph T.M., Unni A.B., Joshy K.S., Kar Mahapatra D., Haponiuk J., Thomas S. (2023). Emerging Bio-Based Polymers from Lab to Market: Current Strategies, Market Dynamics and Research Trends. C.

[199] Filho W.L., Barbir J., Abubakar I.R., Paço A., Stasiskiene Z., Hornbogen M., Christin Fendt M.T., Voronova V., Klõga M. (2022). Consumer Attitudes and Concerns with Bioplastics Use: An International Study. PLoS ONE.

[200] C-Bag. https://Bakeryproduction.Co.Uk/Earth-Wheat-Launch-New-Compostable-Bags-for-Wonky-Bread/.

[201] Eureka. https://www.Packagingdigest.Com/Sustainability/Bread-Company-Goes-Green-Bio-Based-Packaging.

[202] Amerplast. https://amerplast.Com/Products-Packaging-Solutions/Food-Packaging-Labels/Amerbakery/.

[203] BioPack. https://www.biopacktech.com/wholesale-price-clear-pla-biodegradable-bakery-bread-bags-for-sale.html.

[204] BioPack. https://www.Biopacktech.Com/Food-Safe-Self-Adhesive-Home-Compostable-Cellophane-Baking-Bread-Loaf-Bags.Html.

[205] ThePureOption. https://www.Thepureoption.Com/Paper--Pla-Biodegradable-Compostable-Bakery-Bread-Bags-24066-p.Asp.

[206] Mohanty A.K., Wu F., Mincheva R., Hakkarainen M., Raquez J.-M., Mielewski D.F., Narayan R., Netravali A.N., Misra M. (2022). Sustainable Polymers. Nat. Rev. Methods Primers.

[207] Gigante V., Aliotta L., Dal Pont B., Titone V., Botta L., La Mantia F.P., Lazzeri A. (2023). Tailoring Morphology and Mechanical Properties of PLA/PBSA Blends Optimizing the Twin-Screw Extrusion Processing Parameters Aided by a 1D Simulation Software. Polym. Test..

[208] Poyatos-Racionero E., Ros-Lis J.V., Vivancos J.-L., Martínez-Máñez R. (2018). Recent Advances on Intelligent Packaging as Tools to Reduce Food Waste. J. Clean. Prod..

[209] Rodríguez-Parada L., Mayuet P., Gámez A. (2019). Custom Design of Packaging through Advanced Technologies: A Case Study Applied to Apples. Materials.

[210] Rodríguez-Parada L., Mayuet P.F., Gámez A.J. (2019). Evaluation of Reliefs’ Properties on Design of Thermoformed Packaging Using Fused Deposition Modelling Moulds. Materials.

[211] Boz Z., Korhonen V., Koelsch Sand C. (2020). Consumer Considerations for the Implementation of Sustainable Packaging: A Review. Sustainability.

[212] Moshood T.D., Nawanir G., Mahmud F., Mohamad F., Ahmad M.H., AbdulGhani A. (2022). Sustainability of Biodegradable Plastics: New Problem or Solution to Solve the Global Plastic Pollution?. Curr. Res. Green. Sustain. Chem..

[213] Aliotta L., Gigante V., Lazzeri A. (2023). Volcanic Ash as Filler in Biocomposites: An Example of Circular Economy in Volcanic Areas. Sustain. Mater. Technol..

[214] Weinstein J.E., Dekle J.L., Leads R.R., Hunter R.A. (2020). Degradation of Bio-Based and Biodegradable Plastics in a Salt Marsh Habitat: Another Potential Source of Microplastics in Coastal Waters. Mar. Pollut. Bull..

[215] Upasen S., Wattanachai P. (2018). Packaging to Prolong Shelf Life of Preservative-Free White Bread. Heliyon.

[216] Versino F., Ortega F., Monroy Y., Rivero S., López O.V., García M.A. (2023). Sustainable and Bio-Based Food Packaging: A Review on Past and Current Design Innovations. Foods.

[217] Wang Q., Chen W., Zhu W., McClements D.J., Liu X., Liu F. (2022). A Review of Multilayer and Composite Films and Coatings for Active Biodegradable Packaging. NPJ Sci. Food.

[218] Alias A.R., Wan M.K., Sarbon N.M. (2022). Emerging Materials and Technologies of Multi-Layer Film for Food Packaging Application: A Review. Food Control.

[219] Vasile C., Baican M. (2021). Progresses in Food Packaging, Food Quality, and Safety—Controlled-Release Antioxidant and/or Antimicrobial Packaging. Molecules.

[220] Fico D., Rizzo D., De Carolis V., Montagna F., Esposito Corcione C. (2022). Sustainable Polymer Composites Manufacturing through 3D Printing Technologies by Using Recycled Polymer and Filler. Polymer.

[221] Guillard V., Gaucel S., Fornaciari C., Angellier-Coussy H., Buche P., Gontard N. (2018). The Next Generation of Sustainable Food Packaging to Preserve Our Environment in a Circular Economy Context. Front. Nutr..

[222] Feliciano R.J., Guzmán-Luna P., Boué G., Mauricio-Iglesias M., Hospido A., Membré J.-M. (2022). Strategies to Mitigate Food Safety Risk While Minimizing Environmental Impacts in the Era of Climate Change. Trends Food Sci. Technol..

